# The relationship between academic burnout and problematic smartphone use: a three-level meta-analysis

**DOI:** 10.3389/fpsyg.2026.1768092

**Published:** 2026-04-02

**Authors:** Xiaohang Wang, Zainal Bin Madon, Mohamad Salleh Abdul Ghani, Xingfa Long

**Affiliations:** 1 Department of Human Development and Family Studies, Faculty of Human Ecology, Universiti Putra Malaysia, Serdang, Malaysia; 2 Quzhou College of Technology, Quzhou, China

**Keywords:** academic burnout, moderating effect, multilevel meta-analysis, problematic smartphone use, student mental health

## Abstract

**Introduction:**

In recent years, the relationship between academic burnout and problematic smartphone use (PSU) has received increasing attention from researchers. However, existing findings remain inconsistent, with some studies reporting a significant positive association while others finding non-significant results. This study aims to systematically examine the association between academic burnout and PSU and to explore potential moderating factors.

**Methods:**

Following PRISMA guidelines, a comprehensive literature search was conducted across Web of Science, Scopus, PubMed, CNKI, VIP, and Wanfang databases. A total of 79 studies were included, comprising 115 effect sizes and 68,162 participants. A three-level meta-analytic approach was employed to estimate the overall effect size and to examine potential moderators, including demographic characteristics, study contextual features, publication status, and measurement instruments.

**Results:**

The results indicated a moderate positive correlation between academic burnout and PSU (r = 0.438, 95% CI [0.409, 0.467]). Moderator analyses revealed that the relationship was significantly influenced by the year of data collection and the type of PSU measurement instruments used.

**Discussion:**

This study provides a comprehensive and systematic understanding of the association between academic burnout and PSU. The findings offer robust empirical evidence to inform the development of targeted prevention and intervention strategies for PSU.

**Systematic review registration:**

https://doi.org/10.17605/OSF.IO/TRMP3

## 1 Introduction

Smartphones have become deeply integrated into everyday life. They have accelerated communication by removing physical and spatial limitations, enabling users to engage in a wide range of online activities such as virtual meetings, gaming, and other digital services, thereby enhancing efficiency (Hong et al., 2020; Wu et al., 2021). Moreover, their portability allows users to access them anytime and anywhere (Rozgonjuk et al., 2019). However, this convenience may contribute to prolonged usage patterns that potentially disrupt academic responsibilities (Amez and Baert, 2020; Sunday et al., 2021). As a result, the widespread use of smartphones and the issues related to their excessive use have attracted growing interest from researchers worldwide. In current literature, excessive smartphone engagement is frequently conceptualized as “problematic smartphone use,” “smartphone addiction,” or “mobile phone dependence” (Al-Barashdi et al., 2015; Busch and McCarthy, 2021; Hussain et al., 2017; Park et al., 2013; Wang et al., 2018). Although there is no universally agreed-upon definition of problematic smartphone use, the conceptualizations generally fall into two main categories. The first approach frames it as an addictive behavior, drawing upon the concept of “technological addiction” proposed by Griffiths (1995), which is defined as “nonchemical (behavioral) addictions which involve human-machine interaction” (Griffiths, 1995, p. 15). Griffiths (2005) and Billieux et al. (2015a) have suggested that determining whether a behavior qualifies as an addiction involves comparing it to the clinical criteria for substance addiction outlined in the Diagnostic and Statistical Manual of Mental Disorders (American Psychiatric Association, 2013). Conversely, some scholars contest the addiction framework for problematic smartphone use. Critics argue that directly applying substance addiction criteria (e.g., drugs, alcohol, tobacco) to smartphones remains contentious. While users may exhibit substance-like symptoms, these may not indicate physiological dependence on devices themselves (Harris et al., 2020; Panova and Carbonell, 2018). Griffiths (2005) maintains that any behavior exhibiting core addiction features (salience, conflict, tolerance, relapse, mood modification, withdrawal) warrants classification as addiction. Billieux et al. (2015b) critique the overreliance on substance dependence or pathological gambling criteria in smartphone research, arguing that labeling excessive use as addiction oversimplifies complex behavioral phenomena.

Although scholars have discussed the term problematic smartphone use from various perspectives, the latest edition of the DSM-IV (American Psychiatric Association, 2013) does not include diagnostic criteria for smartphone addiction. Therefore, the term “smartphone addiction” should be used with caution in academic research. Scholars argue that problematic smartphone use (PSU) better captures behaviors where individuals continue to use smartphones despite recognizing potential negative consequences (Hao et al., 2021; Harris et al., 2020; Panova and Carbonell, 2018; Roig-Vila et al., 2020). This study focuses on a student population, for whom the severity of smartphone-related issues is generally lower than that seen in addiction. Thus, the term PSU is more appropriate.

Currently, PSU is a widespread phenomenon among students globally. For instance, Liu, Zhou et al. (2022) found that 52.8% of 2,741 Chinese university students exhibited signs of PSU. Similarly, Lee et al. (2023) reported a prevalence rate of 37.1% among 921 adolescents, and Yogesh et al. (2024) found a rate of 64.6% among youths aged 15–19. Spending excessive time on smartphones may lead to various issues, including depression (Alhassan et al., 2018; Ong et al., 2024; Wang et al., 2019), and anxiety (Mayerhofer et al., 2024; Yang X. et al., 2019). These psychological symptoms, in turn, may further contribute to excessive smartphone use (Wang et al., 2019). In school settings, excessive smartphone use can distract students from academic tasks (Troll et al., 2021) and lead to procrastination in completing assignments (Chen and Lyu, 2024; Jin et al., 2024; Yang Z. et al., 2019), ultimately affecting academic performance (Alotaibi et al., 2022; Paterna et al., 2024; Winskel et al., 2019). Therefore, researchers have called for greater attention to the issue of reducing PSU among students (Huang et al., 2021; Hao et al., 2022).

Researchers have explored the influence of various risk and protective factors on PSU (Wong et al., 2024), such as sociocultural, psychological, and personality-related factors (Carvalho et al., 2018; Liu et al., 2023; Long et al., 2024; Wickord and Quaiser-Pohl, 2022). Given that academic tasks are central to students’ lives, research has also begun examining PSU from an academic standpoint (Hao et al., 2021; Hao et al., 2022). Academic burnout refers to a state of emotional exhaustion and detachment from academic activities, often resulting from a lack of motivation or interest in learning despite the obligation to do so (Yang and Lian, 2005). It comprises three dimensions: emotional exhaustion, academic cynicism, and reduced personal accomplishment (Maslach et al., 2001), and is associated with decreased learning motivation, lower satisfaction, and greater risk of health problems (Jacobs et al., 2003). Previous studies have found a significant positive correlation between academic burnout and PSU (Hao et al., 2021; Hao et al., 2022). Students who experience prolonged burnout tend to perform worse academically (Kendall and Castro-Alves, 2018; Madigan and Curran, 2021) and suffer from poorer sleep quality (Qin et al., 2022; Yan et al., 2018). According to the Compensatory Internet Use Theory (CIUT), individuals facing psychosocial difficulties are more likely to engage in PSU as a way to cope with life challenges and negative emotions (Kardefelt-Winther, 2014). PSU can further exacerbate academic burnout (Wang et al., 2023). Grounded in the Job Demands-Resources (JD-R) Model (Zhang et al., 2007), academic burnout occurs when an imbalance arises between high academic demands and low resource availability (Schaufeli and Bakker, 2004). PSU by students also increases their life stress and negative emotions (Al Battashi et al., 2021; Wacks and Weinstein, 2021) and reduces their academic engagement (Li N. et al., 2024). Consequently, fewer resources are available for learning, leading to diminished work resources and thereby increasing susceptibility to academic burnout (Abreu Alves et al., 2022; Wang et al., 2021).

The relationship between academic burnout and problematic smartphone use (PSU) may not always demonstrate statistical significance across student populations and is likely subject to the influence of other variables. Notably, research by Nie (2014) highlights the potential role of dimensional specificity: while the overall association between burnout and PSU scores was non-significant, significant correlations were observed between specific burnout dimensions (emotional exhaustion, academic cynicism) and PSU, as well as between the escape dimension of PSU and overall burnout. Liu and Jin (2018) also found that the low sense of accomplishment dimension of academic burnout was not significantly associated with the avoidance dimension of PSU. This suggests that the apparent inconsistency in findings may stem from focusing on aggregate scores rather than specific facets, or from unaccounted moderating variables.

Given its capacity to directly incorporate and analyze multi-dimensional moderators (Meng et al., 2023), the current study utilizes a three-level meta-analysis to provide a more precise understanding of the burnout-PSU relationship and its underlying moderating mechanisms.

## 2 Potential moderator

The relationship between academic burnout and PSU among university students has shown inconsistent results across studies. These discrepancies may be attributed to differences in participants’ demographic characteristics (e.g., grade level, gender, medical student status), research background characteristics (e.g., sociocultural context, time of data collection, publication status), and measurement-related factors (e.g., measurement instruments).

### 2.1 Grade

Differences in grade level may influence the correlation between academic burnout and PSU. Previous studies have suggested that self-control tends to be lower among individuals under the age of 20 (Bianchi and Phillips, 2005). From a lifespan developmental perspective, executive functioning improves with age (Ferguson et al., 2021), enabling individuals to better regulate their negative experiences (Martin and Ochsner, 2016). When encountering negative emotional states such as academic burnout, older students may be more likely to adopt adaptive coping strategies (Zou, 2019), rather than relying on excessive smartphone use as a means of escape (Wen et al., 2023). Research has shown that the correlation between academic burnout and PSU is higher among secondary school students than university students (Wan, 2020)，likely due to the underdeveloped coping mechanisms of younger students (Jiang et al., 2024). Therefore, this study hypothesizes that grade level is a potential moderator in the relationship between academic burnout and PSU.

### 2.2 Gender

Previous research has suggested that gender may serve as a potential moderating factor in problematic smartphone use (De-Sola Gutiérrez et al., 2016; Hao et al., 2019). Studies have reported that female students tend to spend significantly more time using smartphones than male students (Yang et al., 2021). In addition, compared with males, female adolescents may show greater malleability in self-control and may be more susceptible to social influences, such as smartphone use, when experiencing negative emotions (Park and Lee, 2022).

However, existing findings on the relationship between academic burnout and problematic smartphone use remain inconsistent and show clear gender-related patterns. For example, Tomaszek and Muchacka-Cymerman (2019) found that the positive association between school burnout and problematic internet use was stronger among male adolescents. Similarly, Claesdotter-Knutsson et al. (2021) reported that the relationship between psychological distress and problematic gaming was also stronger in males. Evidence from meta-analytic studies further supports this view. Li et al. (2023), in a meta-analysis examining the association between academic burnout and problematic smartphone use among adolescents and young adults, reported substantial variation in effect sizes across gender groups. In addition, Mao et al. (2024) recommended including gender as a potential moderator in a three-level meta-analysis on the association between problematic internet use and burnout in order to explain between-study heterogeneity. Differences in smartphone use patterns between males and females also provide a possible mechanism for this moderating effect. Male students are more likely to use gaming applications as a form of escapism, whereas female students tend to rely more on multimedia and social networking services to maintain social connections (Chen et al., 2017).

Taken together, these findings suggest that male students experiencing academic burnout may be more likely than female students to develop problematic smartphone use as a coping strategy for academic stress. Based on this evidence, the present study proposes that gender may play a moderating role in the relationship between academic burnout and problematic smartphone use.

### 2.3 Medical student status

Medical students typically face higher academic demands and must invest substantial time and effort to master complex medical knowledge and clinical skills (O’Rourke et al., 2010). Academic burnout is highly prevalent among medical students (Almutairi et al., 2022; Frajerman et al., 2019). Empirical evidence consistently shows that medical students report significantly higher levels of both academic burnout and PSU than their non-medical peers (Ye et al., 2023; Carrard et al., 2025). Comparative studies within health-related disciplines further support the rationale for considering medical student status as a potential moderating variable. An empirical study conducted in Indonesia by Lestari et al. (2026) found that nursing and medical students reported highly comparable levels of academic burnout (nursing students: *M* = 28.69; medical students: *M* = 29.30). No significant differences were observed across the three dimensions of burnout, including emotional exhaustion, cynicism, and professional efficacy. Similarly, a meta-analysis by Gómez-Urquiza et al. (2023) indicated that although nursing students showed lower overall burnout prevalence and lower levels of depersonalization than medical students, their levels of emotional exhaustion were comparable. Studies in allied health fields, such as physical therapy, also report elevated levels of burnout due to the high emotional demands associated with clinical practice (Hwang and Kim, 2022). Taken together, these findings suggest that students in medical-related disciplines share similar characteristics of academic burnout, particularly emotional exhaustion arising from the responsibility of caring for others. This provides empirical support for examining medical student status as a potential moderator in the relationship between academic burnout and problematic smartphone use.

### 2.4 Sociocultural background

Sociocultural contexts significantly shape the coping styles and strategies individuals employ when confronting negative emotions (Matthews et al., 2021). For example, Cabras et al. (2023) found that students in Italy and Russia adopted different coping strategies when dealing with academic burnout. The Interaction of Person-Affect-Cognition-Execution (I-PACE) model posits that individual factors, including cultural background and social environment, play a significant role in the development of PSU (Brand et al., 2019; Brand et al., 2016). Western cultures often encourage emotional expression and self-disclosure, while individuals from Eastern cultures may suppress emotional expression in real-life settings and instead release emotions in virtual spaces, such as through smartphones or the internet, potentially increasing the risk of PSU (Meng et al., 2023; Ying et al., 2016). Therefore, this study hypothesizes that sociocultural background moderates the relationship between academic burnout and PSU, with cultural contexts operationalized through the Social Individualism Index (Hofstede et al., 2010).

### 2.5 Time of data collection

As smartphone usage for entertainment and social interaction has increased over time, the relationship between academic burnout and PSU may have changed. For example, Wang et al. (2023) found that the correlation between these two variables has increased over time. Accordingly, the time of data collection is hypothesized to moderate the relationship between academic burnout and PSU.

### 2.6 Publication status

According to the chronosystem component of ecological systems theory, the developmental trajectory of psychological and behavioral processes must be understood within a temporal context. As smartphone usage for entertainment and social interaction has increased over time, the relationship between academic burnout and PSU may have changed. Wang et al. (2023) found that the correlation between these two variables has increased over time. Accordingly, the time of data collection is hypothesized to moderate the relationship between academic burnout and PSU.

Unpublished studies with non-significant results may be less likely to appear in the literature (Rodgers and Pustejovsky, 2021), potentially introducing publication bias. Therefore, this study assumes that the effect sizes reported in unpublished studies may be smaller than those in published ones, and publication status is considered a potential moderator.

### 2.7 Measurement instruments

Variability in the measurement instruments used across studies may also influence the observed relationship between academic burnout and PSU. Commonly used scales for academic burnout include the Maslach Burnout Inventory–Student Survey (MBI-SS) developed by Schaufeli et al. (2002) and the Learning Burnout Scale (LBS) by Lian et al. (2006). For PSU, widely used instruments include the Mobile Phone Addiction Index (MPAI; Leung, 2008) and the Smartphone Addiction Scale–Short Version (SAS-SV; Kwon et al., 2013). These tools differ in theoretical underpinnings, dimensional structure, and target populations, all of which may impact the strength and direction of the reported correlations. Thus, measurement tools are treated as a potential moderator in this meta-analysis.

## 3 Methods

This meta-analysis has been preregistered on the Open Science Framework (OSF) platform (Registration number: https://doi.org/10.17605/OSF.IO/TRMP3). In addition, the present meta-analysis was conducted in accordance with the Preferred Reporting Items for Systematic Reviews and Meta-Analyses (PRISMA) statement (Moher et al., 2009, 2015).

### 3.1 Literature search

This study conducted a comprehensive search in the following databases: Web of Science, Scopus, PubMed, China National Knowledge Infrastructure (CNKI), VIP, and Wanfang Data. The search strategy was: (“academic burnout” OR “learning burnout” OR “student burnout” OR “school burnout” OR “educational burnout”) AND (“problematic mobile phone use” OR “excessive mobile phone use” OR “compulsive mobile phone use” OR “pathological mobile phone use” OR “mobile phone addiction” OR “mobile phone overuse” OR “mobile phone dependence” OR “problematic smartphone use” OR “smartphone addiction” OR “nomophobia”). The search terms were designed around the constructs of academic burnout and problematic smartphone use. The search was limited to studies published up to September 30, 2024. A total of 327 articles were retrieved.

**Figure 1:**
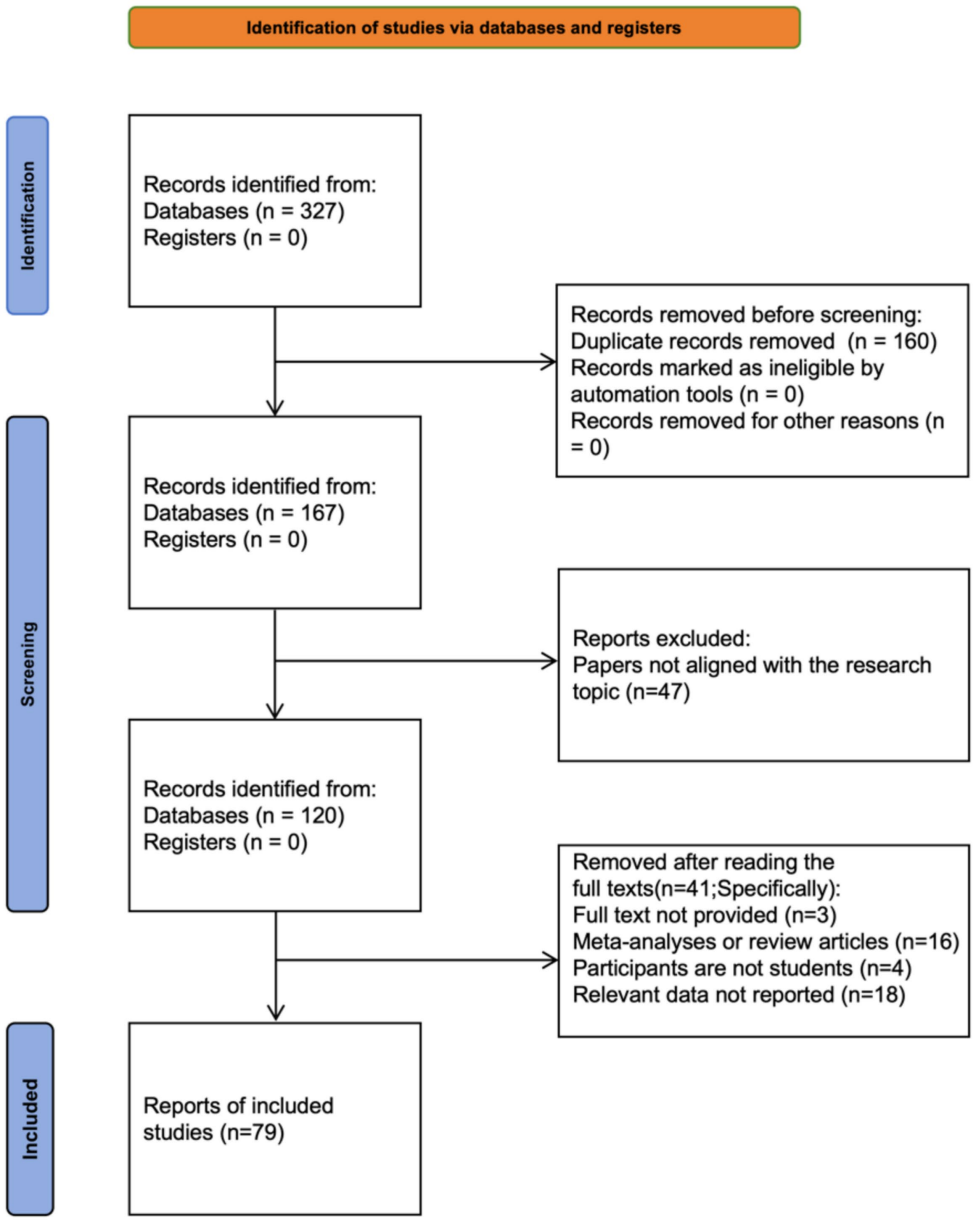
Literature search flowchart.

Studies were included in the meta-analysis based on the following criteria: (1) The article is an empirical study that examined the relationship between academic burnout and PSU. Theoretical papers, literature reviews, and meta-analyses were excluded. The included studies had to provide complete data; (2) The samples across studies were independent. If two studies used the same sample and measured the same variables, only one study was retained. In cases where a dissertation had been published as a journal article, the published version was included; (3) The sample size was clearly reported, and the participants were students from elementary school to university, including primary school, junior high school, high school, and university. Studies involving other populations were excluded; (4) The study reported correlation coefficients (*r*) between academic burnout and PSU, or reported values such as Cohen’s d, t, or F that could be converted to *r* using the formulas provided by Fritz et al. (2012); (5) The studies were published or unpublished before September 30, 2024. In cases of duplicate data, only the earliest version was included; (6) The language of the study was either Chinese or English. The specific process of literature search and screening is shown in Figure 1. Based on the above criteria, the retrieved articles were screened step by step, and finally, 79 studies were included in the meta-analysis.

### 3.2 Coding procedures and quality assessment

The included studies were coded based on the following variables: (1) t year of data collection; (2) the cultural background of participants, which was assessed using Hofstede’s individualism index. Higher scores indicate a higher level of individualism, whereas lower scores reflect a higher level of collectivism (Hofstede et al., 2010); (3) average age of participants; (4) proportion of male participants; (5) grade level, categorized as primary and junior high school, senior high and secondary vocational school, higher vocational college, undergraduate, postgraduate, or mixed samples; (6) whether the sample included medical students; (7) the instrument used to measure academic burnout; (8) the instrument used to measure PSU. During the coding process, the following principles were applied: (1) Each independent sample was coded once. If a study reported multiple independent samples, each sample was coded separately; (2) If effect sizes were reported separately according to participant characteristics (e.g., male/female), they were coded independently; (3) If a study included multiple measurement indicators of the variables, effect sizes were coded for each indicator separately.

Subsequently, the quality of each included study was assessed based on the criteria of the Quality Assessment Tool for Observational Cohort and Cross-Sectional Studies developed by the National Institutes of Health (NIH). Each criterion was scored as 1 if met and 0 if not met (or not applicable) (Meng et al., 2023). According to the total score, study quality was categorized as good (total score > 7), fair (total score 5–7), or poor (total score < 5). The detailed quality assessment for all included studies is provided in Supplementary Table S1. Higher scores indicate better study quality.

To minimize subjectivity in the coding process, the first author initially conducted independent coding and developed a coding manual to standardize the procedures. Another graduate student in psychology then performed independent coding. Any discrepancies between the two coders were discussed collectively until consensus was reached. Two researchers independently coded the data, and the inter-rater reliability reached a Kappa coefficient of 0.93, indicating a high level of agreement between coders (Landis and Koch, 1977).

### 3.3 Calculation of effect sizes

This study used the correlation coefficient as the effect size index and extracted or calculated each reported correlation between academic burnout and PSU from the included studies. All correlation coefficients were converted into Fisher’s z scores for the calculation of the overall and moderating effects (Cooper et al., 2019). According to the criteria of Cohen (1992) correlation coefficients of 0.10, 0.30, and 0.50 were considered small, medium, and large effect sizes, respectively.

### 3.4 Model selection

Most primary studies included in this meta-analysis reported multiple effect sizes derived from the same sample, resulting in statistical dependencies among these effect sizes (Cheung, 2014). Traditional meta-analytic approaches, such as fixed-effects or random-effects models, may inflate the precision of pooled estimates due to unaddressed dependencies (Lipsey and Wilson, 2001). To address the issue of effect size dependency, many studies have adopted the three-level meta-analysis approach to handle multiple effect sizes reported within the same study and to enhance statistical power (Meng et al., 2023). Therefore, this study applied a three-level random-effects model to examine the overall effect, heterogeneity, moderation effects, and publication bias.

### 3.5 Heterogeneity and moderation analyses

The three-level meta-analytic model identifies three distinct sources of variance: (1) sample variance of the effect sizes (Level 1), (2) variance between effect sizes extracted from the same study (Level 2), and (3) variance between studies (Level 3) (Cheung, 2014). Heterogeneity was assessed using the *Q* test. In addition, one-tailed log likelihood ratio tests were conducted on the level 2 and level 3 variances to further determine the distribution of heterogeneity (Gao et al., 2024). When heterogeneity was present, *I*^2^ values of 25, 50, and 75% were interpreted as low, moderate, and high levels of heterogeneity, respectively, based on the criteria proposed by Higgins et al. (2003). Further moderator analyses were conducted to explore the potential sources of heterogeneity. Moderators in this study included both continuous and categorical variables. Continuous moderators were: (1) the proportion of male participants in the sample, (2) the average age of participants, (3) the year the data were collected, and (4) the individualism index of the participants’ cultural context. Categorical moderators were: (1) educational stage of the participants, (2) whether the participants were medical students, (3) the instrument used to assess academic burnout, (4) the instrument used to assess problematic smartphone use, and (5) publication status.

### 3.6 Publication bias and sensitivity analysis

Publication bias refers to the tendency for studies with significant findings to be more likely published (Rodgers and Pustejovsky, 2021), This bias may limit the representativeness of published studies for the overall body of completed research, thus reducing the reliability of meta-analytic results (Franco et al., 2014). To mitigate this bias, the present meta-analysis incorporated both published journal articles and unpublished dissertations and conference papers.

Compared to conventional publication bias detection methods, Egger’s multilevel meta-analytic (MLMA) regression provides enhanced control over Type I errors when analyzing dependent effect sizes (Rodgers and Pustejovsky, 2021). Given that most studies included in this meta-analysis reported multiple correlated effect sizes, Egger’s MLMA regression was selected to evaluate publication bias. If Egger’s regression is significant (*p* < 0.05) or the funnel plot shows asymmetry, the trim-and-fill method is applied to estimate the number of missing studies needed to achieve symmetry (Duval and Tweedie, 2004), If *R*_0_^+^ > 3, *L*_0_^+^ > 2, publication bias is considered present (Fernández-Castilla et al., 2021).

### 3.7 Data analysis

All analyses were conducted using the *metafor* package in R version 4.3.0 (Viechtbauer, 2010). The restricted maximum likelihood method was used to estimate model parameters (Viechtbauer, 2005). A two-tailed *p*-value of less than 0.05 was considered statistically significant.

## 4 Results

### 4.1 Overall effect size

A total of 79 studies were included in this meta-analysis, yielding 115 effect sizes and involving 68,162 participants. Among them, 62 were journal articles and 17 were theses or dissertations. The literature included was published up to September 30, 2024. Basic information of the included studies is presented in Table 1.

**Table 1:** Characteristics of original studies included in the meta-analysis.

Number	Study name (First author, year)	*k*	Survey year	N (sample size)	*r* (effect size)	Country	Individualism index (IDV)	Mean age	Gender (% male)	Sample type	Medical students (Y/N)	Academic burnt measurement	PSU measurement	Publication type (Journal/Thesis)
1	Bai et al. (2020)	1	2018	1794	0.168	China	43	12.6	0.51	Primary and secondary school students	N	MBI-SS	MPAI	Journal Article
2	Cheng and Zhang (2020)	1	NA	673	0.510	China	43	NA	NA	Postgraduate students	N	ABQ-GS	MPAI	Journal Article
3	Chen et al. (2023)	1	NA	2,110	0.274	China	43	NA	0.51	Undergraduate students	N	LBS	MPDQ	Journal Article
4	Hao et al. (2021)	1	2020	748	0.348	China	43	20.12	0.24	Undergraduate students	N	LBS	SAS-SV	Journal Article
5	Hao et al. (2022)	1	2020	766	0.244	China	43	20.1	0.26	Undergraduate students	N	LBS	SAS-SV	Journal Article
6	Hu et al. (2024)	1	NA	628	0.429	China	43	27.62	0.38	Undergraduate students	Y	LBS	MPATS	Journal Article
7	Jiang et al. (2024)	1	NA	828	0.510	China	43	16.79	0.34	High School students	N	ASBI	MPATS	Journal Article
8	Jin et al. (2024)	1	2023	930	0.547	China	43	NA	0.29	Undergraduate students	N	MBI-SS	MPDIS	Journal Article
9	Kaya (2024)	1	NA	403	0.438	Turkey	46	16.024	0.41	High school students	N	SBS	SAS-SV	Journal Article
10	Li W. et al. (2024)	1	NA	1,253	0.420	China	43	18.78	0.46	Vocational college students	N	LBS	MPATS	Journal Article
11	Li et al. (2021)	1	2020	2077	0.503	China	43	16.27	0.14	Adolescents	N	LBS	SRQ-APMPU	Journal Article
12	Liu et al. (2023)	2	2022	1,564	0.520	China	43	19.14	0.55	Undergraduate students	N	LBS	SAS-C	Journal Article
			2023	1,564	0.500	China	43	19.14	0.55	Undergraduate students	N	LBS	SAS-C	Journal Article
13	Qin et al. (2020)	1	NA	964	0.400	China	43	20.03	NA	Undergraduate students	N	LBS	MPATS	Journal Article
14	Samek et al. (2024)	1	NA	132	0.200	United States	60	18.8	0.47	Undergraduate students	N	MBI-SS	SABAS	Journal Article
15	Wang et al. (2023)	2	2019	2,260	0.390	China	43	12.67	0.50	Middle school students	N	MBI-SS	SAS-SV	Journal Article
			2020	2,260	0.400	China	43	12.67	0.50	Middle school students	N	MBI-SS	SAS-SV	Journal Article
16	Yang et al. (2024)	1	NA	752	0.301	China	43	19.36	0.34	Undergraduate students	N	MBI-SS	MPAI	Journal Article
17	Yao et al. (2025)	1	2023	810	0.390	China	43	14.56	0.48	Adolescents	N	ASBI	SAS-SV	Journal Article
18	Ye et al. (2023)	1	2022	2,948	0.473	China	43	NA	0.25	Undergraduate students	Mixed	LBS	SAS	Journal Article
19	Zhang C. et al. (2021)	2	NA	771	0.356	China	43	19.87	0.36	Undergraduate students	N	LBS	MPATS	Journal Article
			NA	704	0.405	China	43	24.68	0.28	Postgraduate students	N	LBS	MPATS	Journal Article
20	Zhang C.-H et al. (2021)	1	NA	1,062	0.368	China	43	19.52	0.40	Undergraduate and vocational college students	Y	LBS	MPATS	Journal Article
21	Zhang H. et al. (2023)	1	2020	1,256	0.360	China	43	20.09	0.32	Undergraduate students	Mixed	LBS	GSP	Journal Article
22	Zhang et al. (2024)	1	2022	3,190	0.435	China	43	21.6	0.38	Undergraduate students	Y	LBS	SAS-SV	Journal Article
23	Zhou et al. (2022)	1	2020	1,445	0.431	China	43	19.65	NA	Undergraduate students	Y	LBS	SAS-SV	Journal Article
24	Zhu et al. (2023)	1	2022	823	0.421	China	43	18.55	0.61	Undergraduate students	N	MBI-SS	SAS-SV	Journal Article
25	Wan (2020)	4	NA	537	0.440	China	43	NA	NA	Middle school students	N	ASBI	MPAI	Thesis
			NA	621	0.380	China	43	NA	NA	Undergraduate students	N	ASBI	MPAI	Thesis
			2017	602	0.350	China	43	17.07	NA	Secondary school students and university students	N	ASBI	MPAI	Thesis
			2018	602	0.370	China	43	17.07	NA	Secondary school students and university students	N	ASBI	MPAI	Thesis
26	He et al. (2022)	1	NA	1,191	0.410	China	43	17.38	0.51	Secondary school students and university students	N	ASBI	MPAI	Journal Article
27	Yu et al. (2022)	1	2020	196	0.440	China	43	NA	0.15	Undergraduate students	Y	LBS	MPATS	Journal Article
28	Nong (2022)	1	NA	786	0.517	China	43	NA	0.38	Undergraduate students	N	LBS	MPAI	Journal Article
29	Feng and Tao (2019)	1	NA	704	0.466	China	43	NA	0.46	Undergraduate students	N	LBS	SAS-C	Journal Article
30	Liu J. et al. (2022)	1	2020	239	0.463	China	43	NA	0.36	Undergraduate students	Y	LBS	MPATS	Journal Article
31	Liu et al. (2019)	1	NA	881	0.450	China	43	20.39	0.50	Undergraduate students	N	ASBI	MPATS	Journal Article
32	Liu et al. (2021)	1	NA	323	0.471	China	43	21.61	0.22	Undergraduate students	N	ASBI	MPATS	Journal Article
33	Lu (2017)	1	2016	364	0.363	China	43	NA	0.52	Middle school students	N	ASBI	MPDS-MSS	Journal Article
34	Wu et al. (2022)	1	2020	883	0.474	China	43	NA	0.24	Undergraduate students	Mixed	LBS	MPATS	Journal Article
35	Zhou (2021)	1	NA	592	0.450	China	43	NA	0.22	Undergraduate and vocational college students	N	LBS	MPAI	Thesis
36	Cui (2023)	1	NA	591	0.500	China	43	NA	0.53	Middle school students	N	ASBI	MPAI	Thesis
37	Zhang W. et al. (2023)	1	NA	619	0.277	China	43	NA	0.10	Associate degree students	Y	LBS	MPAI	Journal Article
38	Zhang and Shen (2015)	1	NA	218	0.404	China	43	NA	0.54	Vocational college students	N	LBS	MPATS	Journal Article
39	Zhang et al. (2019)	1	NA	239	0.348	China	43	NA	0.68	Undergraduate students	Y	LBS	MPAI	Journal Article
40	Zhang F. et al. (2020)	1	2019	910	0.442	China	43	NA	0.54	Undergraduate students	N	LBS	SAS-C	Journal Article
41	Zhang Y. et al. (2020)	1	2017	635	0.338	China	43	19.21	0.39	Undergraduate students	N	LBS	SAS	Journal Article
42	Zhang (2017)	1	NA	459	0.484	China	43	16.82	0.41	High school students	N	ASBI	MPAI	Thesis
43	Zhang (2021)	1	NA	3,090	0.385	China	43	NA	0.39	Undergraduate and associate degree students	Y	LBS	MPATS	Thesis
44	Qu et al. (2017)	1	NA	582	0.399	China	43	20.89	0.23	Vocational college students	N	LBS	MPATS	Journal Article
45	Cao (2018)	1	NA	193	0.348	China	43	NA	0.45	Undergraduate students	N	LBS	SAS-C	Journal Article
46	Li B. et al. (2022)	1	NA	1,505	0.600	China	43	13.66	0.51	Middle school students	N	ASBI	MPAI	Journal Article
47	Li C. et al. (2022)	1	2020	511	0.463	China	43	20.25	0.23	Undergraduate students	N	LBS	MPATS	Journal Article
48	Liang (2019)	1	NA	807	0.209	China	43	NA	0.28	Vocational school and higher vocational college students	Y	ASBI	MPATS	Thesis
49	Shen (2017)	1	NA	218	0.404	China	43	NA	0.54	Higher vocational college students	N	LBS	MPATS	Journal Article
50	Wang et al. (2020)	1	NA	388	0.673	China	43	NA	0.44	Undergraduate and graduate students	N	LBS	MPATS	Thesis
51	Cheng (2021)	1	2019	885	0.402	China	43	20.63	0.26	Undergraduate students	N	LBS	MPAI	Journal Article
52	Cheng (2019)	1	NA	673	0.510	China	43	NA	0.50	Postgraduate students	N	QPAB	MPAI	Thesis
53	Nie (2014)	1	NA	352	0.103	China	43	18.41	0.29	Secondary vocational school students	N	ASBI	MPAI	Thesis
54	Hu (2022)	1	NA	576	0.470	China	43	NA	0.48	Middle school students	N	MSABQ	MPAI	Thesis
55	Ge (2013)	1	NA	211	0.305	China	43	16.86	0.76	Secondary vocational school students	N	ASBI	MPATS	Journal Article
56	Jiang et al. (2017)	1	NA	308	0.450	China	43	NA	0.57	Undergraduate students	N	ASBI	MPPUS	Journal Article
57	Xue et al. (2022)	1	NA	373	0.539	China	43	NA	0.43	Undergraduate students	Mixed	LBS	MPAI	Journal Article
58	Yuan and Ma (2024)	1	2022	1,097	0.556	China	43	NA	NA	Higher vocational college students	N	LBS	SAS-C	Journal Article
59	Zhao (2024)	1	2023	1,027	0.330	China	43	NA	0.49	Secondary school students	N	ASBI	SAS-SV	Thesis
60	Deng (2021)	1	NA	296	0.330	China	43	NA	0.66	Secondary vocational school students	N	LBS	MPAI	Journal Article
61	Zou (2018)	1	NA	316	0.237	China	43	NA	0.41	Middle school students	N	ASBI	MPDS-MSS	Thesis
62	Lu and Zhou (2019)	1	NA	1,095	0.379	China	43	17.05	0.05	Vocational college students	Y	LBS	MPATS	Journal Article
63	Lu (2023)	1	NA	1,418	0.641	China	43	NA	0.76	Undergraduate and graduate students	N	LBS-PCS	SAS-C	Thesis
64	Chen et al. (2022)	1	NA	1791	0.470	China	43	NA	0.24	Undergraduate and associate degree students	N	LBS	SAS-C	Journal Article
65	Chen et al. (2024)	1	NA	483	0.476	China	43	NA	0.18	Undergraduate students	N	LBS	MPATS	Journal Article
66	Chen et al. (2021)	1	NA	812	0.470	China	43	NA	0.35	Undergraduate students	Y	LBS	MPATS	Journal Article
67	Chen (2019)	1	NA	872	0.440	China	43	NA	0.74	Secondary vocational school students	N	LBS-SVSS	SAS-C	Thesis
68	Li Q. et al. (2022)	1	NA	290	0.230	China	43	NA	0.40	Undergraduate students	N	LBS	MPAI	Journal Article
69	Wei et al. (2023)	1	2022	1,345	0.497	China	43	NA	0.11	Undergraduate students	Y	LBS	SAS-SV	Journal Article
70	Gu et al. (2021)	1	NA	389	0.481	China	43	NA	0.38	Undergraduate students	Mixed	LBS	MPATS	Journal Article
71	Ma et al. (2020)	1	2019	357	0.430	China	43	20.05	0.10	Undergraduate students	Y	LBS	MPAI	Journal Article
72	Ma (2019)	1	NA	274	0.514	China	43	NA	0.39	Secondary vocational school students	N	ASBI	SAS-C	Thesis
73	Huang and Zhou (2016)	1	NA	274	0.410	China	43	NA	0.42	Undergraduate students	N	LBS	MPDQ-US	Journal Article
74	Ye (2021)	1	NA	312	0.548	China	43	NA	0.49	Primary and secondary school students	N	ASBI	MPAI	Thesis
75	Li et al. (2020)	3	NA	825	0.290	China	43	20.12	0.25	Undergraduate students	Y	LBS	MPAI	Journal Article
			NA	825	0.250	China	43	20.12	0.25	Undergraduate students	Y	LBS	MPAI	Journal Article
			NA	825	0.100	China	43	20.12	0.25	Undergraduate students	Y	LBS	MPAI	Journal Article
76	Liu and Jin (2018)	12	NA	397	0.390	China	43	NA	0.56	Undergraduate students	N	LBS	MPAI	Journal Article
			NA	397	0.320	China	43	NA	0.56	Undergraduate students	N	LBS	MPAI	Journal Article
			NA	397	0.220	China	43	NA	0.56	Undergraduate students	N	LBS	MPAI	Journal Article
			NA	397	0.390	China	43	NA	0.56	Undergraduate students	N	LBS	MPAI	Journal Article
			NA	397	0.320	China	43	NA	0.56	Undergraduate students	N	LBS	MPAI	Journal Article
			NA	397	0.240	China	43	NA	0.56	Undergraduate students	N	LBS	MPAI	Journal Article
			NA	397	0.100	China	43	NA	0.56	Undergraduate students	N	LBS	MPAI	Journal Article
			NA	397	0.330	China	43	NA	0.56	Undergraduate students	N	LBS	MPAI	Journal Article
			NA	397	0.200	China	43	NA	0.56	Undergraduate students	N	LBS	MPAI	Journal Article
			NA	397	0.150	China	43	NA	0.56	Undergraduate students	N	LBS	MPAI	Journal Article
			NA	397	0.090	China	43	NA	0.56	Undergraduate students	N	LBS	MPAI	Journal Article
			NA	397	0.190	China	43	NA	0.56	Undergraduate students	N	LBS	MPAI	Journal Article
77	Yu et al. (2023)	4	NA	182	0.169	China	43	NA	0.27	Undergraduate students	N	LBS	SMPDS	Journal Article
			NA	182	0.385	China	43	NA	0.27	Undergraduate students	N	LBS	SMPDS	Journal Article
			NA	182	0.598	China	43	NA	0.27	Undergraduate students	N	LBS	SMPDS	Journal Article
			NA	182	0.334	China	43	NA	0.27	Undergraduate students	N	LBS	SMPDS	Journal Article
78	Cheng et al. (2018)	4	2017	607	0.321	China	43	NA	0.39	Vocational college students	N	LBS	MPAI	Journal Article
			2017	607	0.277	China	43	NA	0.39	Vocational college students	N	LBS	MPAI	Journal Article
			2017	607	0.209	China	43	NA	0.39	Vocational college students	N	LBS	MPAI	Journal Article
			2017	607	0.272	China	43	NA	0.39	Vocational college students	N	LBS	MPAI	Journal Article
79	Shi (2023)	12	NA	550	0.363	China	43	NA	0.51	Postgraduate students	N	QPAB	MPAI	Thesis
			NA	550	0.381	China	43	NA	0.51	Postgraduate students	N	QPAB	MPAI	Thesis
			NA	550	0.313	China	43	NA	0.51	Postgraduate students	N	QPAB	MPAI	Thesis
			NA	550	0.313	China	43	NA	0.51	Postgraduate students	N	QPAB	MPAI	Thesis
			NA	550	0.357	China	43	NA	0.51	Postgraduate students	N	QPAB	MPAI	Thesis
			NA	550	0.397	China	43	NA	0.51	Postgraduate students	N	QPAB	MPAI	Thesis
			NA	550	0.328	China	43	NA	0.51	Postgraduate students	N	QPAB	MPAI	Thesis
			NA	550	0.266	China	43	NA	0.51	Postgraduate students	N	QPAB	MPAI	Thesis
			NA	550	0.310	China	43	NA	0.51	Postgraduate students	N	QPAB	MPAI	Thesis
			NA	550	0.323	China	43	NA	0.51	Postgraduate students	N	QPAB	MPAI	Thesis
			NA	550	0.262	China	43	NA	0.51	Postgraduate students	N	QPAB	MPAI	Thesis
			NA	550	0.238	China	43	NA	0.51	Postgraduate students	N	QPAB	MPAI	Thesis

Academic burnout measures: MBI-SS = Maslach Burnout Inventory-Student Survey; LBS = Learning Burnout Scale for Undergraduates; ABQ-GS = Academic Burnout Questionnaire for Graduate Students; ASBI = Adolescent Student Burnout Inventory; SBS = School Burnout Scale; QPAB = Questionnaire of Postgraduates’ Academic Burnout; MSABQ = Middle School Students’ Academic Burnout Questionnaire; LBS-PCS = Learning Burnout Scale for Police Colleges Students; LBS-SVSS = Self-Developed Learning Burnout Scale for Secondary Vocational School Students. Problematic smartphone use measures: MPAI = Mobile Phone Addiction Index; MPDQ = Mobile Phone Dependence Questionnaire; SAS-SV = Smartphone Addiction Scale-Short Version; MPATS = Mobile Phone Addiction Tendency Scale; MPDIS = Chinese Version of the Mobile Phone Dependence Index Scale; SRQ-APMPU = Self-Rating Questionnaire for Adolescent Problematic Mobile Phone Use; SAS-C = Smartphone Addiction Scale for College Students; GSP = Generic Scale of Phubbing; MPDS-MSS = Mobile Phone Dependency Scale of Middle School Students; SABAS = Six-Item Smartphone Application-Based Addiction Scale; SAS = Smartphone Addiction Scale; MPPUS = Mobile Phone Problem Usage Scale; MPDQ-US = Self-Developed Mobile Phone Dependence Questionnaire for University Students; SMPDS = Self-Developed Mobile Phone Dependence Scale.

**Figure 2:**
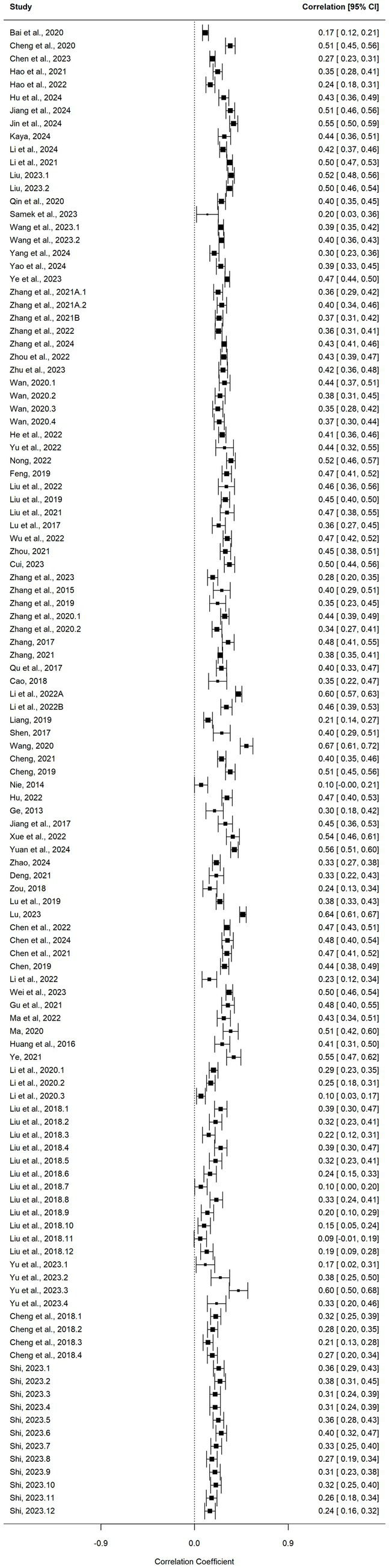
Forest plot for the relation between academic burnout and PSU.

A three-level meta-analytic model was used to estimate the main effect between academic burnout and PSU. Results revealed a statistically significant positive correlation between academic burnout and PSU (*r* = 0.438, *p* < 0.001, 95%CI[0.409, 0.467]). According to Cohen (1992), this correlation represents a medium effect size. The forest plots of individual and overall effect sizes are presented in Figure 2.

### 4.2 Heterogeneity analysis

To assess the heterogeneity of the overall variance, a *Q* test was conducted. The *Q* value for the three-level meta-analysis model was 1523.782 (*p* < 0.001), indicating significant heterogeneity in the results. Further analysis using one-tailed log likelihood ratio tests was conducted to examine the distribution of heterogeneity. The results showed that the variance of effect sizes within the same study (Level 2, σ^2^ = 0.005, *p* < 0.001, I2 = 28.950%) and the variance between different studies (Level 3, σ^2^ = 0.011, *p* < 0.001, I2 = 63.352%) were both significant. Based on the criteria of Higgins et al. (2003), there was moderate heterogeneity within studies and substantial heterogeneity between studies. Therefore, moderator analysis was necessary to further explain the relationship between academic burnout and problematic smartphone use.

### 4.3 Publication bias test and sensitivity analysis

**Figure 3:**
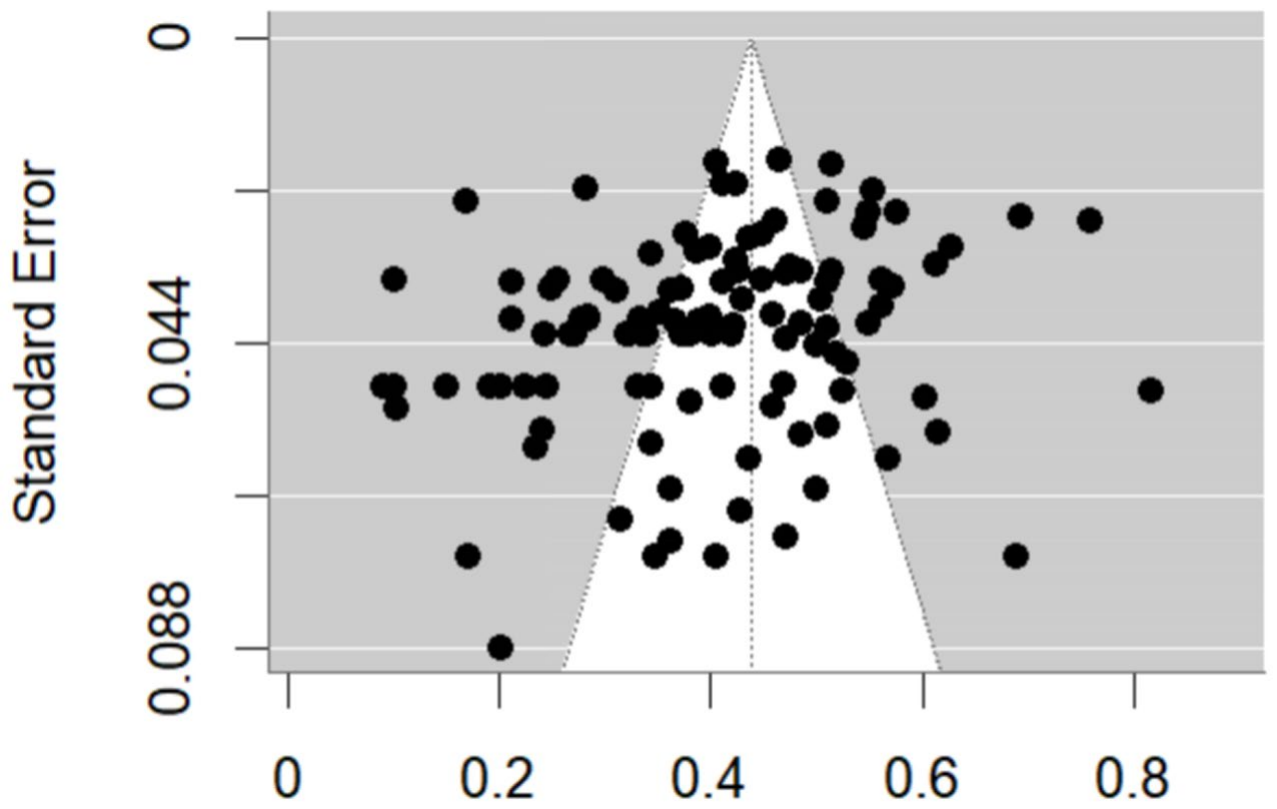
Funnel plot of effect size distribution.

The funnel plot is presented in Figure 3. The effect sizes were not uniformly and symmetrically distributed above the midline, suggesting potential publication bias. Therefore, Egger-MLMA regression was conducted and found to be non-significant (*z* = −1.598, *p* = 0.110), with an intercept of −16.298, 95% CI [−36.280, 3.682], indicating no significant publication bias in this meta-analysis.

Trim-and-fill analysis further supported this result, with *R*_0_^+^ = 1 and *L*_0_^+^ = 0, both below the thresholds (*R*_0_^+^ > 3, *L*_0_^+^ > 2), suggesting no substantial missing studies and thus minimal publication bias.

A leave-one-out sensitivity analysis was conducted by removing each effect size and re-estimating the model. The results showed that the main effect of academic burnout on PSU use remained significant in all cases, with correlation coefficients ranging from 0.434 to 0.442. These findings indicate that the current meta-analytic results are robust and reliable.

### 4.4 Analysis of moderators

Meta-regression was conducted to examine whether demographic variables (grade, proportion of male participants, medical student status), study characteristics (individualism index, data collection year), publication type (thesis vs. journal article), and measurement instruments (for academic burnout and PSU) moderated the relationship between academic burnout and PSU. Among demographic variables, no significant moderating effects were found for grade (*F* = 0.306, *p* = 0.908), proportion of male participants (*F* = 0.167, *p* = 0.684), or medical student status (*F* = 1.136, *p* = 0.325).

Among study characteristics, the year of data collection significantly moderated the relationship (*F* (1, 31) = 8.940, *p* < 0.05), with the correlation between academic burnout and PSU use increasing over time (*β* = 0.026, *p* = 0.005). No significant moderation effects were found for the individualism index (*F* = 2.153, *p* = 0.145) or publication status (*F* = 0.616, *p* = 0.434).

Regarding measurement tools, the type of instrument used to assess PSU showed a significant moderating effect (*F* (1, 110) = 2.551, *p* < 0.05). Specifically, studies using the SAS-C reported a stronger correlation (*r* = 0.494, *p* < 0.05). However, the measurement tool for academic burnout did not significantly moderate the relationship (*F* = 1.228, *p* = 0.303). Detailed results are presented in Table 2.

**Table 2:** Moderator analysis of the relationship between academic burnout and problematic smartphone use.

Moderator	*k*	Intercept/mean *z* (95% CI)	*β* (95% CI)	Mean *r*	*t*	*F*	*p*	Level 2	Level 3
Demographic variables									
Grade						0.306	0.908	0.005	0.012
Primary and junior high	7	0.469 (0.360, 0.577)***		0.437 (0.345, 0.519)	8.559				
High school and secondary Vocational student	8	0.424 (0.326, 0.522)***	−0.045 (−0.191, 0.101)	0.399 (0.315, 0.479)	8.567				
Higher vocational student	11	0.409 (0.315, 0.503)***	−0.060 (−0.204, 0.084)	0.387 (0.305, 0.464)	8.603				
Undergraduate students	58	0.432 (0.391, 0.473)***	−0.037 (−0.153, 0.080)	0.407 (0.372, 0.440)	20.834				
Postgraduate students	15	0.466 (0.348, 0.584)***	−0.003 (−0.163, 0.158)	0.435 (0.335, 0.523)	7.818				
Mixed samples	14	0.461 (0.389, 0.533)***	−0.008 (−0.138, 0.123)	0.429 (0.370, 0.487)	12.665				
Proportion of males	107	0.435 (0.404, 0.466)***	0.041(−0.159,0.241)	-	0.408	0.167	0.684	0.006	0.011
Medical student status						1.136	0.325	0.005	0.0109
Medical students	17	0.405 (0.337, 0.472)***	-	0.384 (0.326, 0.440)	11.934				
Non-medical students	93	0.441 (0.408, 0.475)***	0.037 (−0.039, 0.112)	0.414 (0.387, 0.441)	26.009				
Mixed sample	5	0.504 (0.387, 0.621)***	0.100 (−0.035, 0.234)	0.464 (0.368, 0.552)	8.554				
Research background characteristics									
Sociocultural context	115	0.438 (0.409,0.468)***	−0.013(−0.031, 0.005)	-	−1.467	2.153	0.145	0.005	0.011
Data collection year	33	0.429 (0.392, 0.467)***	0.026 (0.008, 0.044)**	-	2.990	8.940	0.005	0.000	0.008
Publication status						0.616	0.434	0.005	0.0111
Journal articles	84	0.432 (0.399, 0.465)***		0.407 (0.379, 0.433)	−0.785				
Theses/Dissertations	31	0.460 (0.398, 0.523)***	0.028 (−0.043, 0.099)	0.430 (0.378, 0.479)	0.785				
Measurement factors									
Academic burnout scale						1.228	0.303	0.005	0.011
LBS	69	0.436 (0.399, 0.473)***		0.410 (0.379, 0.440)	23.212				
ASBI	21	0.437 (0.375, 0.499)***	0.001 (−0.071, 0.073)	0.410 (0.358, 0.460)	13.965				
QPAB	13	0.425 (0.260, 0.590)***	−0.011 (−0.180, 0.158)	0.401 (0.254, 0.531)	5.1102				
MBI-SS	7	0.369 (0.264, 0.475)***	−0.067 (−0.179, 0.045)	0.354 (0.258, 0.443)	6.9347				
Others	4	0.556 (0.426, 0.687)***	0.120 (−0.015, 0.256)	0.505 (0.403, 0.597)					
PSU scale						2.551	0.043	0.005	0.009
MPAI	55	0.402 (0.353, 0.450)***		0.381 (0.340, 0.421)	16.490				
MPATS	24	0.461 (0.408, 0.514)***	0.016 (−0.075, 0.108)	0.430 (0.387, 0.473)	17.267				
SAS-SV	11	0.418 (0.341, 0.495)***	0.060 (−0.012, 0.131)	0.396 (0.329, 0.456)	10.716				
SAS-C	10	0.543 (0.461, 0.626)***	0.142 (0.046, 0.237)**	0.494 (0.430, 0.555)	13.017				
Others	15	0.410 (0.337, 0.482)***	0.008 (−0.079, 0.095)	0.388 (0.326, 0.447)	11.186				

Level 2 variance refers to within-study variance; Level 3 variance refers to between-study variance. k = number of effect sizes; mean z = Fisher’s z-transformed effect size; CI = confidence interval; β = regression coefficient from meta-regression; r = Pearson correlation coefficient; df = degrees of freedom. LBS = Learning Burnout Scale for Undergraduates; ASBI = Adolescent Student Burnout Inventory; QPAB = Questionnaire of Postgraduates’ Academic Burnout; MBI-SS = Maslach Burnout Inventory-Student Survey; MPAI = Mobile Phone Addiction Index; MPATS = Mobile Phone Addiction Tendency Scale; SAS-SV = Smartphone Addiction Scale-Short Version; SAS-C = Smartphone Addiction Scale for College Students. *p < 0.05; **p < 0.01;***p < 0.001.

## 5 Discussion

### 5.1 The relationship between academic burnout and PSU

This study employed a three-level meta-analysis to examine the relationship between academic burnout and PSU. The main effect analysis revealed a significant positive correlation between the two variables, with a moderate effect size. This finding provides broader empirical support for the link between academic burnout and PSU use among students.

The result aligns with the CIUT, which posits that PSU serves as a maladaptive coping strategy to escape from real-life stressors and negative emotions by seeking emotional relief in virtual environments (Kardefelt-Winther, 2014). Previous studies have shown that academic burnout is associated with individuals’ negative emotions (Cheng et al., 2020). When students experience these emotions, they may turn to the internet for temporary relief (Kardefelt-Winther, 2014). Ideally, students facing academic difficulties or emotional distress due to high demands should adopt more adaptive coping strategies (Guo et al., 2025). However, given the ubiquity of smartphones and their capacity to provide instant gratification, students may tend to overuse them as a way to manage their burnout, which in turn increases the likelihood of problematic use. This finding is consistent with previous research (Wang et al., 2023). Consistent with the Job Demands-Resources (JD-R) model posits that any form of work requires a balance between job demands and job resources. When job demands increase and job resources decrease, individuals are more likely to experience burnout (Schaufeli and Bakker, 2004). In the context of education, students’ academic responsibilities can be conceptualized as their work tasks within the academic environment (Schaufeli et al., 2002). PSU may reduce students’ available cognitive and emotional resources for academic engagement. This reduction in perceived academic resources can increase the likelihood of academic burnout (Zhang H. et al., 2023; Zhou et al., 2022). Furthermore, when students devote a substantial amount of time to escapism or entertainment activities through smartphones, they may gradually lose interest in real-life academic tasks, which can further contribute to disengagement and emotional exhaustion (Chen et al., 2023). Students with severe PSU are often found to lag behind their peers academically (Hawi and Samaha, 2016). As a result, they may need to invest even more effort to catch up. However, PSU can easily distract attention (Feng et al., 2019), leading to decreased motivation and enthusiasm for learning (Hu et al., 2024), which may in turn lead to a decline in academic enthusiasm and motivation, as well as the development of negative attitudes toward learning (Hu et al., 2024). Over time, these negative academic experiences may increase the risk of academic burnout (Wang et al., 2023; Zhu et al., 2023).

The significant variance observed at both the within-study (Level 2) and between-study (Level 3) levels indicates heterogeneity in the main effect. This suggests that the relationship between academic burnout and PSU cannot be interpreted in isolation (Harrer et al., 2021). Contextual factors may influence this association. Therefore, it is necessary to investigate potential moderating variables to explain the observed heterogeneity and gain a deeper understanding of the relationship between the two constructs.

### 5.2 Moderating effects on the relationship between academic burnout and PSU

This study identified the measurement tool for PSU as a significant moderator in the relationship between academic burnout and PSU. Specifically, studies using the SAS-C reported significantly higher correlation coefficients than those using other instruments. This may be attributed to the cultural context in which the scale was developed. Both the MPATS and SAS-C were designed based on Chinese college student samples. However, the SAS-C incorporated insights from smartphone application addiction research in addition to traditional internet addiction frameworks, resulting in a more comprehensive measurement across multiple dimensions (Su et al., 2014). In contrast, the SAS-SV was developed in Korea for Korean students (Kwon et al., 2013). Moreover, the SAS-C was specifically tailored to university students, enhancing its precision in measuring the association between academic burnout and PSU.

The year of data collection also emerged as a significant moderator. The increasing accessibility of smartphones and rising ownership rates have expanded opportunities for ubiquitous smartphone use, including contexts such as classroom settings or driving (Billieux et al., 2015a), which may further exacerbate patterns of problematic usage. This trend may also reflect the increasing academic and psychological pressure faced by students in recent years (Clabaugh et al., 2021; Von Keyserlingk et al., 2022). When students experience excessive stress without adequate relief, they may turn to smartphone-based social platforms for immediate distraction and emotional release (Ducasse et al., 2017). Thus, smartphones have evolved from mere communication devices into tools for managing negative emotions. Furthermore, the COVID-19 pandemic has led to prolonged online learning, which increases feelings of isolation, stress, and distraction (Xu et al., 2020), while reduced social support and interaction may heighten the risk of academic burnout (Wang et al., 2020). All of these factors may contribute to the strengthening of the relationship between academic burnout and PSU.

Other moderators, however, were found to be non-significant. Demographic variables, including grade, gender, and whether the sample consisted of medical students, did not significantly moderate the relationship. Prior research has similarly shown that age and gender do not play significant roles in this relationship (Hu et al., 2024; Jiang et al., 2024). Although PSU has been shown to negatively impact both mental health and academic performance among medical students (Rozgonjuk et al., 2018; Zhong et al., 2022), the current meta-analysis included only a small number of studies specifically targeting this population. Interestingly, the highest effect size was found in studies with mixed samples of medical and non-medical students, suggesting that continued attention should be paid to the mental health and academic performance of medical students (Leow et al., 2023). It is also worth considering the nature of study, that is, the academic discipline or training context, as a potential factor in explaining the observed patterns, even though medical student status did not show a significant moderating effect. For students in helping professions such as medicine and nursing, the core of their training involves responding to the suffering and needs of others. This professional orientation requires students to engage in continuous emotional labor and empathic engagement from the early stages of clinical training. Such unique stressors may increase the risk of compassion fatigue and emotional exhaustion. As a result, the pathway from academic burnout to problematic smartphone use may become stronger, with smartphone use functioning as a form of immediate relief or maladaptive coping through avoidance (Wolotira, 2023; Gómez-Urquiza et al., 2023).

Empirical comparisons within health-related disciplines further support this interpretation. For example, nursing students and medical students have been found to report highly similar levels of overall burnout (Lestari et al., 2026). Students in allied health professions, such as physical therapy, also demonstrate elevated levels of burnout due to the demands associated with clinical training (Hwang and Kim, 2022). These findings suggest that the nature of study or training context may influence both burnout levels and coping strategies, which in turn may affect students’ problematic smartphone use. Future research should therefore pay closer attention to academic burnout and problematic smartphone use among students in these disciplines.

The moderating effect of sociocultural background was not statistically significant. However, this finding should be interpreted with caution because the cultural representation of the included studies was highly imbalanced. Specifically, only two studies involved participants from countries other than China, namely Türkiye and the United States (Kaya, 2024; Samek et al., 2024). This uneven distribution of samples substantially limited the statistical power to detect cross-cultural moderating effects and also restricted the external validity of the findings. Therefore, the present results should not be generalized to different cultural contexts. Future meta-analytic research should include studies from a wider range of cultural backgrounds in order to more comprehensively examine potential sociocultural moderating effects.

Publication status was also not a significant moderator. Meta-analyses strive for comprehensive coverage of available studies. Despite extensive database searches in the current study, some unpublished works may have been difficult to retrieve. Future research should aim to collect a more complete and systematic body of literature to enhance the robustness of the findings. Finally, the type of measurement tool used for academic burnout did not significantly moderate the relationship. The most commonly used instruments in the included studies were the Student Learning Burnout Scale developed by Lian et al. (2006) and Wu et al. (2007), and the Maslach Burnout Inventory–Student Survey (MBI-SS) developed by Schaufeli et al. (2002).

These tools were grounded in the conceptual and three-dimensional model of occupational burnout proposed by Maslach and Leiter (1997). Although the samples used in scale development differed, these tools comprehensively capture the construct of academic burnout, which may explain the non-significant moderating effect of the measurement instrument.

### 5.3 Limitation and future directions

This study employed a three-level meta-analysis to synthesize empirical findings on the relationship between academic burnout and PSU. It also examined potential moderators influencing this relationship. First, the results support the assumptions of the CIUT. Secondly, data collection time and the measurement tool for PSU were the key moderators influencing the relationship between academic burnout and PSU. This indicates that the strength of this association is shaped not only by methodological characteristics of the studies but also by temporal variations, consistent with Bronfenbrenner’s ecological systems theory. Finally, this study incorporated samples from diverse cultural backgrounds, enhancing the cultural breadth of the analysis. This provides a valuable foundation for future cross-cultural research.

This study also offers several practical implications. The significant association between academic burnout and PSU indicates that reducing academic burnout may reduce PSU among students. First, families and educational institutions can mitigate academic burnout and reduce problematic smartphone use by cultivating supportive environments and strengthening social support systems (Ye et al., 2021). Second, mental health professionals should pay attention to the potential risk of problematic smartphone use in students experiencing academic burnout. Helping students adopt more adaptive coping strategies when facing academic difficulties and negative emotions may reduce the likelihood of problematic smartphone behavior (Ma et al., 2022). Third, implementing targeted interventions such as group therapy and mindfulness programs (Liu F. et al., 2022; Tang et al., 2021) can enhance academic motivation, alleviate burnout, and lower risks of problematic smartphone use. These strategies collectively address both academic burnout and its behavioral consequences.

This study has several aspects that warrant further improvement. First, the data were based on self-reports from participants, suggesting that future research should incorporate additional assessment methods to more accurately examine the relationship between academic burnout and problematic smartphone use. Second, the cultural representativeness of the included studies was limited. Most of the studies included in this meta-analysis were conducted in China, while only a small number were carried out in other cultural contexts. This imbalance in the sample distribution restricts the cross-cultural generalizability and external validity of the findings. Therefore, the conclusions of this meta-analysis should primarily be interpreted within the context of China or similar cultural settings. Future research should include more culturally diverse samples in order to better examine potential cross-cultural differences in the relationship between academic burnout and problematic smartphone use. Finally, previous research has identified psychological traits such as resilience and anxiety as relevant factors influencing the relationship between academic burnout and problematic smartphone use (Hao et al., 2021; Hao et al., 2022; Jiang et al., 2024). Future investigations should further expand the range of moderating variables to deepen the understanding of this relationship.

## 6 Conclusion

This study employed a three-level meta-analytic approach to reveal a significant positive correlation between academic burnout and problematic smartphone use. This relationship was moderated by the time of data collection and the measurement instruments used to assess problematic smartphone use. Specifically, studies employing the SAS-C reported significantly stronger correlations between academic burnout and PSU compared to studies using other instruments. In contrast, factors such as participants’ grade, gender, medical student status, sociocultural background, publication status, and the measurement tools for academic burnout did not significantly moderate the relationship between academic burnout and PSU. It should be noted that the non-significant moderating effect of sociocultural background should be interpreted with caution, as most of the included studies were conducted in China, which limits the cross-cultural representativeness of the findings. This study contributes to a deeper understanding of the impact of academic burnout on PSU and offers valuable insights for the development of prevention and intervention strategies targeting problematic smartphone behavior.

## Data Availability

The original contributions presented in the study are included in the article/Supplementary material, further inquiries can be directed to the corresponding author/s.

## References

[ref1] Abreu Alves S. Sinval J. Lucas Neto L. Marôco J. Gonçalves Ferreira A. Oliveira P. (2022). Burnout and dropout intention in medical students: the protective role of academic engagement. BMC Med. Educ. 22:83. doi: 10.1186/s12909-021-03094-9, 35130892 PMC8821797

[ref2] Al Battashi N. Al Omari O. Sawalha M. Al Maktoumi S. Alsuleitini A. Al Qadire M. (2021). The relationship between smartphone use, insomnia, stress, and anxiety among university students: a cross-sectional study. Clin. Nurs. Res. 30, 734–740. doi: 10.1177/1054773820983161, 33375850

[ref3] Al-Barashdi H. S. Bouazza A. Jabur N. H. (2015). Smartphone addiction among university undergraduates: a literature review. J. Sci. Res. Rep. 4, 210–225. doi: 10.9734/JSRR/2015/12245

[ref4] Alhassan A. A. Alqadhib E. M. Taha N. W. Alahmari R. A. Salam M. Almutairi A. F. (2018). The relationship between addiction to smartphone usage and depression among adults: a cross sectional study. BMC Psychiatry 18:148. doi: 10.1186/s12888-018-1745-4, 29801442 PMC5970452

[ref5] Almutairi H. Alsubaiei A. Abduljawad S. Alshatti A. Fekih-Romdhane F. Husni M. . (2022). Prevalence of burnout in medical students: a systematic review and meta-analysis. Int. J. Soc. Psychiatry 68, 1157–1170. doi: 10.1177/00207640221106691, 35775726

[ref6] Alotaibi M. S. Fox M. Coman R. Ratan Z. A. Hosseinzadeh H. (2022). Smartphone addiction prevalence and its association on academic performance, physical health, and mental well-being among university students in umm Al-Qura University (UQU), Saudi Arabia. Int. J. Environ. Res. Public Health 19:3710. doi: 10.3390/ijerph19063710, 35329397 PMC8954621

[ref7] American Psychiatric Association (2013). Diagnostic and Statistical Manual of mental Disorders: DSM-5™. 5th Edn. Arlington, VA: American Psychiatric Publishing, Inc.

[ref8] Amez S. Baert S. (2020). Smartphone use and academic performance: a literature review. Int. J. Educ. Res. 103:101618. doi: 10.1016/j.ijer.2020.101618

[ref9] Bai C. Chen X. Han K. (2020). Mobile phone addiction and school performance among Chinese adolescents from low-income families: a moderated mediation model. Child Youth Serv. Rev. 118:105406. doi: 10.1016/j.childyouth.2020.105406

[ref10] Bianchi A. Phillips J. G. (2005). Psychological predictors of problem mobile phone use. Cyberpsychol. Behav. 8, 39–51. doi: 10.1089/cpb.2005.8.39, 15738692

[ref11] Billieux J. Maurage P. Lopez-Fernandez O. Kuss D. J. Griffiths M. D. (2015a). Can disordered mobile phone use be considered a behavioral addiction? An update on current evidence and a comprehensive model for future research. Curr. Addict. Rep. 2, 156–162. doi: 10.1007/s40429-015-0054-y

[ref12] Billieux J. Philippot P. Schmid C. Maurage P. De Mol J. Van der Linden M. (2015b). Is dysfunctional use of the mobile phone a behavioural addiction? Confronting symptom-based versus process-based approaches. Clin. Psychol. Psychother. 22, 460–468. doi: 10.1002/cpp.1910, 24947201

[ref13] Brand M. Wegmann E. Stark R. Müller A. Wölfling K. Robbins T. W. . (2019). The interaction of person-affect-cognition-execution (I-PACE) model for addictive behaviors: update, generalization to addictive behaviors beyond internet-use disorders, and specification of the process character of addictive behaviors. Neurosci. Biobehav. Rev. 104, 1–10. doi: 10.1016/j.neubiorev.2019.06.032, 31247240

[ref14] Brand M. Young K. S. Laier C. Wölfling K. Potenza M. N. (2016). Integrating psychological and neurobiological considerations regarding the development and maintenance of specific internet-use disorders: an interaction of person-affect-cognition-execution (I-PACE) model. Neurosci. Biobehav. Rev. 71, 252–266. doi: 10.1016/j.neubiorev.2016.08.033, 27590829

[ref15] Busch P. A. McCarthy S. (2021). Antecedents and consequences of problematic smartphone use: a systematic literature review of an emerging research area. Comput. Hum. Behav. 114:106414. doi: 10.1016/j.chb.2020.106414

[ref16] Cabras C. Konyukhova T. Lukianova N. Mondo M. Sechi C. (2023). Gender and country differences in academic motivation, coping strategies, and academic burnout in a sample of Italian and Russian first-year university students. Heliyon 9:e16617. doi: 10.1016/j.heliyon.2023.e16617, 37260901 PMC10227335

[ref17] Cao M. (2018). Mobile phone addiction and its relationship with learning burnout of local colleges and universities of college students. Chin. J. Health Psychol. 26, 953–956. doi: 10.13342/j.cnki.cjhp.2018.06.041

[ref18] Carrard V. Bourquin C. Berney S. Bart P. A. Bodenmann P. Berney A. (2025). Comparison of mental health and burnout between medical and nonmedical students. PLoS One 20:e0328145. doi: 10.1371/journal.pone.0328145, 41066345 PMC12510498

[ref19] Carvalho L. F. Sette C. P. Ferrari B. L. (2018). Problematic smartphone use relationship with pathological personality traits: systematic review and meta-analysis. Cyberpsychol. J. Psychosoc. Res. Cyberspace 12:5. doi: 10.5817/CP2018-3-5

[ref20] Chen Y. (2019) The effect of time management tendency on learning burnout of secondary vocational school students: the mediating role of mobile phone dependence. Master's thesis, Fujian Normal University

[ref21] Chen X. Chen M. Wang Y. Long J. Dong P. (2021). Study on relationship between mobile phone addiction and learning burnout of college students. China Educ. Technol. Equip. 2, 26–28, 34. doi: 10.3969/j.issn.1671-489X.2021.02.026.

[ref22] Chen S. Li Z. Ruan X. Lv S. (2024). The relationship between professional identity and negative implicit absenteeism behavior among university students. Beijing Educ. 8, 73–76.

[ref23] Chen B. Liu F. Ding S. Ying X. Wang L. Wen Y. (2017). Gender differences in factors associated with smartphone addiction: a cross-sectional study among medical college students. BMC Psychiatry 17:341. doi: 10.1186/s12888-017-1503-z, 29017482 PMC5634822

[ref24] Chen G. Lyu C. (2024). The relationship between smartphone addiction and procrastination among students: a systematic review and meta-analysis. Pers. Individ. Differ. 224:112652. doi: 10.1016/j.paid.2024.112652

[ref25] Chen C. Shen Y. Xiao F. Ni J. Zhu Y. (2023). The effect of smartphone dependence on learning burnout among undergraduates: the mediating effect of academic adaptability and the moderating effect of self-efficacy. Front. Psych. 14:1155544. doi: 10.3389/fpsyt.2023.1155544, 37736057 PMC10509764

[ref26] Chen Y. Song E. Jin J. (2022). Mediation model analysis of future time perception and learning burnout. J. Bioeduc. 10, 276–280.

[ref27] Cheng Y. (2019) Research on the Influence of mobile phone addiction on academic burnout in postgraduate students: the mediating role of sleep quality and the moderating role of anxiety. Master’s thesis, Jinan University

[ref28] Cheng G. (2021). The relationship between family socioeconomic status and college students’ learning burnout——multiple mediating effects of boredom and mobile phone dependence. Psychol. Mon. 16, 11–13. doi: 10.19738/j.cnki.psy.2021.05.005

[ref29] Cheng J. Guo K. Yan J. (2018). A study on the relationship between smartphone addiction and academic burnout among students in higher vocational colleges. J. Campus Life Ment. Health 16, 414–418.

[ref30] Cheng J. Zhao Y Y Wang J, & and Sun Y. H. (2020). Academic burnout and depression of Chinese medical students in the pre-clinical years: the buffering hypothesis of resilience and social support. Psychol. Health Med., 25, 1094–1105. doi: 10.1080/13548506.2019.1709651 31887065

[ref31] Cheng Y. Zhang Z. (2020). A study on the influence of Mobile phone addiction on academic burnout in postgraduate students with mediating effect of sleep quality. E3S Web Conf. 218:04019. doi: 10.1051/e3sconf/202021804019

[ref32] Cheung M. W.-L. (2014). Modeling dependent effect sizes with three-level meta-analyses: a structural equation modeling approach. Psychol. Methods 19, 211–229. doi: 10.1037/a0032968, 23834422

[ref33] Clabaugh A. Duque J. F. Fields L. J. (2021). Academic stress and emotional well-being in United States college students following onset of the COVID-19 pandemic. Front. Psychol. 12:628787. doi: 10.3389/fpsyg.2021.628787, 33815214 PMC8010317

[ref34] Claesdotter-Knutsson E. André F. Fridh M. Delfin C. Hakansson A. Lindström M. (2021). Gender-based differences and associated factors surrounding excessive smartphone use among adolescents: cross-sectional study. JMIR Pediatrics Parenting 4:e30889. doi: 10.2196/30889, 34813492 PMC8663478

[ref35] Cohen J. (1992). Statistical power analysis. Curr. Dir. Psychol. Sci. 1, 98–101. doi: 10.1111/1467-8721.ep10768783

[ref36] Cooper H. Hedges L. V. Valentine J. C. (2019). The Handbook of Research Synthesis and meta-Analysis. New York: Russell Sage Foundation.

[ref37] Cui Y. (2023) Study on the Relationship between academic burnout and interpersonal sensitivity among middle school students: mobile phone dependence as the intermediary. Master’s thesis, Bohai University

[ref38] Deng C. (2021). A study on the relationship between mobile phone dependence, academic burnout, and sense of achievement among secondary vocational school students. J. Vocat. Educ. 11, 44–48.

[ref39] De-Sola Gutiérrez J. Rodríguez de Fonseca F. Rubio G. (2016). Cell-phone addiction: a review. Front. Psych. 7:175. doi: 10.3389/fpsyt.2016.00175, 27822187 PMC5076301

[ref40] Ducasse D. Holden R. R. Boyer L. Artero S. Calati R. Guillaume S. . (2017). Psychological pain in suicidality: a meta-analysis. J. Clin. Psychiatry 78:16r10732. 10.4088/JCP.16r10732 28872267

[ref41] Duval S. Tweedie R. (2004). Trim and fill: a simple funnel-plot–based method of testing and adjusting for publication bias in meta-analysis. Biometrics 56, 455–463. doi: 10.1111/j.0006-341X.2000.00455.x, 10877304

[ref42] Feng Q. Tao W. (2019). The relationships among mobile phone dependence, self-objectification, and academic burnout in university students. Jiangxi Soc. Sci. 39, 245–253.

[ref43] Feng S. Wong Y. K. Wong L. Y. Hossain L. (2019). The internet and Facebook usage on academic distraction of college students. Comput. Educ. 134, 41–49. doi: 10.1016/j.compedu.2019.02.005

[ref44] Ferguson H. J. Brunsdon V. E. A. Bradford E. E. F. (2021). The developmental trajectories of executive function from adolescence to old age. Sci. Rep. 11:1382. doi: 10.1038/s41598-020-80866-1, 33446798 PMC7809200

[ref45] Fernández-Castilla B. Lies D. Laleh J. Natasha B. S. Patrick O. den Van Noortgate W. (2021). Detecting selection bias in meta-analyses with multiple outcomes: a simulation study. J. Exp. Educ. 89, 125–144. doi: 10.1080/00220973.2019.1582470

[ref46] Frajerman A. Morvan Y. Krebs M.-O. Gorwood P. Chaumette B. (2019). Burnout in medical students before residency: a systematic review and meta-analysis. Eur. Psychiatry 55, 36–42. doi: 10.1016/j.eurpsy.2018.08.006, 30384110

[ref47] Franco A. Malhotra N. Simonovits G. (2014). Publication bias in the social sciences: unlocking the file drawer. Science 345, 1502–1505. doi: 10.1126/science.1255484, 25170047

[ref48] Fritz C. O. Morris P. E. Richler J. J. (2012). Effect size estimates: current use, calculations, and interpretation. J. Exp. Psychol. Gen. 141, 2–18. doi: 10.1037/a0024338, 21823805

[ref49] Gao S. Yu D. Assink M. Chan K. L. Zhang L. Meng X. (2024). The association between child maltreatment and pathological narcissism: a three-level meta-analytic review. Trauma Violence Abuse 25, 275–290. doi: 10.1177/15248380221147559, 36651026

[ref51] Ge X. (2013). An investigation of the relationship between mobile phone addiction tendency and academic burnout among secondary vocational students. Mental Health Educ. Primary Secondary School 15, 14–17.

[ref52] Gómez-Urquiza J. L. Velando-Soriano A. Membrive-Jiménez M. J. Ramírez-Baena L. Aguayo-Estremera R. Ortega-Campos E. . (2023). Prevalence and levels of burnout in nursing students: a systematic review with meta-analysis. Nurse Educ. Pract. 72:103753. doi: 10.1016/j.nepr.2023.103753, 37651959

[ref53] Griffiths M. (1995). Technological addictions. Clin. Psychol. Forum 76, 14–19.

[ref54] Griffiths M. (2005). A ‘components’ model of addiction within a biopsychosocial framework. J. Subst. Use 10, 191–197. doi: 10.1080/14659890500114359

[ref55] Gu J. Quan Q. Zhang J. (2021). Impact of mobile phone addiction of college students on sleep quality and learning burnout. J. North China Univ. Sci. Technol. 23, 389–394.

[ref56] Guo S. Zou X. Tao Y. Lv Y. Liu X. Huang S. (2025). Gender differences in symptom interactions between problematic smartphone use and social anxiety in adolescents: a network analysis. Child Adolesc. Psychiatry Ment. Health 19:9. doi: 10.1186/s13034-025-00865-w, 39953549 PMC11829345

[ref57] Hao Z. Jin L. Huang J. Lyu R. Cui Q. (2021). Academic burnout and problematic smartphone use during the COVID-19 pandemic: the effects of anxiety and resilience. Front. Psych. 12:725740. doi: 10.3389/fpsyt.2021.725740, 34744819 PMC8564350

[ref58] Hao Z. Jin L. Huang J. Wu H. (2022). Stress, academic burnout, smartphone use types and problematic smartphone use: the moderation effects of resilience. J. Psychiatr. Res. 150, 324–331. doi: 10.1016/j.jpsychires.2022.03.019, 35447526

[ref59] Hao Z. Jin L. Li Y. Akram H. R. Saeed M. F. Ma J. . (2019). Alexithymia and mobile phone addiction in Chinese undergraduate students: the roles of mobile phone use patterns. Comput. Hum. Behav. 97, 51–59. doi: 10.1016/j.chb.2019.03.001

[ref60] Harrer M. Cuijpers P. Furukawa T. Ebert D. (2021). Doing Meta-analysis with R: A Hands-on Guide. Boca Raton, FL: Chapman and Hall/CRC.

[ref61] Harris B. Regan T. Schueler J. Fields S. A. (2020). Problematic mobile phone and smartphone use scales: a systematic review. Front. Psychol. 11:672. doi: 10.3389/fpsyg.2020.00672, 32431636 PMC7214716

[ref62] Hawi N. S. Samaha M. (2016). To excel or not to excel: strong evidence on the adverse effect of smartphone addiction on academic performance. Comput. Educ. 98, 81–89. doi: 10.1016/j.compedu.2016.03.007

[ref63] He A. Wan J. Hui Q. (2022). The relationship between mobile phone dependence and mental health among adolescents: the mediating role of academic burnout and the moderating role of coping styles. Psychol. Dev. Educ. 38, 391–398. doi: 10.16187/j.cnki.issn1001-4918.2022.03.10

[ref65] Higgins J. P. T. Thompson S. G. Deeks J. J. Altman D. G. (2003). Measuring inconsistency in meta-analyses. BMJ 327, 557–560. doi: 10.1136/bmj.327.7414.557, 12958120 PMC192859

[ref66] Hofstede G. Hofstede G. Minkov M. (2010). Cultures and Organizations: Software of the mind. 3rd Edn. New York, NY: McGraw Hill.

[ref67] Hong W. Liu R.-D. Ding Y. Zhen R. Jiang R. Fu X. (2020). Autonomy need dissatisfaction in daily life and problematic mobile phone use: the mediating roles of boredom proneness and mobile phone gaming. Int. J. Environ. Res. Public Health 17:5305. doi: 10.3390/ijerph17155305, 32717969 PMC7432443

[ref68] Hu S. (2022) A study on the relationship between junior high school students' mobile phone dependence and academic burnout: a chain mediation effect analysis. Master’s thesis, Mudanjiang Normal University

[ref69] Hu Q. Ningling Y. Qi H. Cong C. Lei X. Xingjing G. . (2024). Mobile phone addiction and psychological capital mediates the relationship between life satisfaction and learning burnout in Chinese medical postgraduate students: a structural equation model analysis. Psychol. Res. Behav. Manag. 17, 3169–3180. doi: 10.2147/PRBM.S466422, 39296529 PMC11408269

[ref70] Huang S. Lai X. Xue Y. Zhang C. Wang Y. (2021). A network analysis of problematic smartphone use symptoms in a student sample. J. Behav. Addict. 9, 1032–1043. doi: 10.1556/2006.2020.00098, 33372911 PMC8969737

[ref71] Huang Y. Zhou J. (2016). A study on the relationship between the use of mobile phones and academic burnout among college students. J. Shijiazhuang Univ. 18, 139–142.

[ref72] Hussain Z. Griffiths M. D. Sheffield D. (2017). An investigation into problematic smartphone use: the role of narcissism, anxiety, and personality factors. J. Behav. Addict. 6, 378–386. doi: 10.1556/2006.6.2017.052, 28849667 PMC5700726

[ref73] Hwang E. Kim J. (2022). Factors affecting academic burnout of nursing students according to clinical practice experience. BMC Med. Educ. 22:346. doi: 10.1186/s12909-022-03422-7, 35524256 PMC9077997

[ref74] Jacobs S. R. Dodd D. Jacobs S. R. Dodd D. (2003). Student burnout as a function of personality, social support, and workload. J. Coll. Stud. Dev. 44, 291–303. doi: 10.1353/csd.2003.0028

[ref75] Jiang W. Li Y. Zhang J. Cheng H. Yin Q. Xu L. . (2017). A study on the relationship between improper mobile phone use and academic burnout among university students. Sci. Technol. Vision 30, 26–17.

[ref76] Jiang W. Liu S. Liu M. Zhang C. Chong Z. Y. Xu W. (2024). The relationship between mindfulness and academic burnout in senior high school students during COVID-19 pandemic: the chain mediating role of social anxiety and smartphone addiction tendency. Curr. Psychol. 43, 33658–33667. doi: 10.1007/s12144-024-06101-6

[ref77] Jin C. Fan C. Niu J. (2024). How physical exercise influences academic burnout among Chinese “double non” college students: the chain mediation role of mobile phone addiction and learning engagement. Front. Psychol. 14:1289499. doi: 10.3389/fpsyg.2023.1289499, 38250123 PMC10797110

[ref78] Kardefelt-Winther D. (2014). A conceptual and methodological critique of internet addiction research: towards a model of compensatory internet use. Comput. Hum. Behav. 31, 351–354. doi: 10.1016/j.chb.2013.10.059

[ref79] Kaya B. (2024). Smartphone addiction and psychological wellbeing among adolescents: the multiple mediating roles of academic procrastination and school burnout. Br. J. Guid. Couns. 52, 815–829. doi: 10.1080/03069885.2024.2304208

[ref80] Kendall M. C. Castro-Alves L. J. (2018). Tool for predicting medical student burnout from sustained stress levels. J. Osteopathic Med. 118, 364–365. doi: 10.7556/jaoa.2018.036, 29809250

[ref81] Kwon M. Kim D.-J. Cho H. Yang S. (2013). The smartphone addiction scale: development and validation of a short version for adolescents. PLoS One 8:e83558. doi: 10.1371/journal.pone.0083558, 24391787 PMC3877074

[ref82] Landis J. R. Koch G. G. (1977). The measurement of observer agreement for categorical data. Biometrics 33, 159–174. doi: 10.2307/2529310, 843571

[ref83] Lee K. Ching S. Ali N. Ooi C. Kamal S. Amat A. . (2023). Prevalence and factors associated with smartphone addiction among adolescents–a nationwide study in Malaysia. Int. J. Ment. Health Promot. 25, 237–247. doi: 10.32604/ijmhp.2023.013407

[ref84] Leow M. Q. H. Chiang J. Chua T. J. X. Wang S. Tan N. C. (2023). The relationship between smartphone addiction and sleep among medical students: a systematic review and meta-analysis. PLoS One 18:e0290724. doi: 10.1371/journal.pone.0290724, 37713408 PMC10503710

[ref85] Lestari D. R. Rahmawati R. D. Rahmayanti D. Yusuf A. Fitryasari R. P. K. Hidayat T. . (2026). Academic burnout: a comparative study between nursing and medical students in South Kalimantan, Indonesia. Gac. Med. Caracas 134, S49–S59. doi: 10.47307/GMC.2026.134.S1.7

[ref86] Leung L. (2008). Linking psychological attributes to addiction and improper use of the mobile phone among adolescents in Hong Kong. J. Child. Media 2, 93–113. doi: 10.1080/17482790802078565

[ref87] Li N. Fu L. Yang H. Zhao W. Wang X. Yan Y. . (2024). The relationship between mobile phone dependence and academic burnout in Chinese college students: a moderated mediator model. Front. Psych. 15:1382264. doi: 10.3389/fpsyt.2024.1382264, 38827446 PMC11140007

[ref88] Li Y. Jia X. Lv J. Li J. Su H. Yu H. (2020). A study on the relationship between smartphone dependence and academic burnout among medical students: the mediating role of academic engagement and emotions. China Higher Med. Educ. 17, 11–12.

[ref89] Li S. Li M. Wang C. Wang Y. (2023). The more academic burnout students got, the more problematic mobile phone use they suffered? A meta-analysis of mainland Chinese adolescents and young adults. Front. Psychol. 13:1084424. doi: 10.3389/fpsyg.2022.1084424, 36726513 PMC9885163

[ref90] Li C. Ma P. He B. (2022). The relationship between boredom and learning burnout: the mediating role of mobile phone addiction. Psychol. Mon. 17, 30–32. doi: 10.19738/j.cnki.psy.2022.07.009

[ref91] Li Q. Sui H. Luo M. Zhao C. Chen Y. (2022). A study on the relationship between mobile phone dependence, psychological capital, and academic burnout among university students. Sci. Res. 11, 8–11.

[ref92] Li Y. Sun Q. Sun M. Sun P. Sun Q. Xia X. (2021). Physical exercise and psychological distress: the mediating roles of problematic Mobile phone use and learning burnout among adolescents. Int. J. Environ. Res. Public Health 18:9261. doi: 10.3390/ijerph18179261, 34501851 PMC8430986

[ref93] Li W. Xu T. Diao L. Wu Q. (2024). The impact of perceived discrimination on Mobile phone addiction among Chinese higher vocational college students: a chain mediating role of negative emotions and learning burnout. Psychol. Res. Behav. Manag. 17, 401–411. doi: 10.2147/PRBM.S440958, 38343428 PMC10854227

[ref94] Li B. Zhang Y. Yang Q. Ye B. Wang S. Li Z. . (2022). The relationships of academic self-handicapping on junior student’s Mobile phone addiction: a moderated mediation model. Chin. J. Clin. Psychol. 30, 310–313. doi: 10.16128/j.cnki.1005-3611.2022.02.013

[ref95] Lian R. Yang L. Wu L. (2006). A study on the professional commitment and learning burnout of undergraduates and their relationship. Psychol. Sci. 29, 47–51. doi: 10.16719/j.cnki.1671-6981.2006.01.013

[ref96] Liang R. (2019) Investigation on the status of mobile addiction, boredom, and learning burnout in a Secondary Vocational School in Hohhot. Master’s thesis, Jilin University

[ref97] Lipsey M. W. Wilson D. B. (2001). Practical Meta-Analysis. Thousand Oaks, CA: Sage Publications, Inc.

[ref99] Liu S. Jin C. (2018). The relationship between college students’ mobile phone addiction and learning burnout: personality as a moderator. Chin. J. Spec. Educ., 5, 86–91.

[ref100] Liu C. Ren L. Rotaru K. Liu X. Li K. Yang W. . (2023). Bridging the links between big five personality traits and problematic smartphone use: a network analysis. J. Behav. Addict. 12, 128–136. doi: 10.1556/2006.2022.00093, 36763335 PMC10260210

[ref101] Liu J. Wang T. Hou N. Li X. Huang L. (2022). The relationship between self-efficacy and academic burnout of medical students: the mediating role of mobile phone addiction. Psychological Monthly 17, 46–47. doi: 10.19738/j.cnki.psy.2022.01.016

[ref102] Liu X. Ye C. Wu M. (2021). Research on mobile phone addiction and learning burnout. Psychological Monthly 16:26.

[ref103] Liu Y. Ye B. Yang Q. (2019). Stressful life events on student burnout in college students: a chain mediation analysis. Chin. J. Clin. Psychol. 27, 782–784. doi: 10.16128/j.cnki.1005-3611.2019.04.029

[ref104] Liu F. Zhang Z. Liu S. Feng Z. (2022). Effectiveness of brief mindfulness intervention for college students’ problematic smartphone use: the mediating role of self-control. PLoS One 17:e0279621. doi: 10.1371/journal.pone.0279621, 36548308 PMC9778502

[ref106] Long J. Liu Y. Wang Y. Pottié A. Cornil A. Deleuze J. . (2024). The mediating effects of perceived family support in the relationship between anxiety and problematic smartphone use: a cross-cultural validation. J. Nerv. Ment. Dis. 212, 76–83. doi: 10.1097/NMD.0000000000001738, 38030146

[ref107] Lu C. (2017). A study on relationship among hope, mobile phone dependence and learning burnout in rural left-behind children. J. Longyuan Univ. 35, 129–136. doi: 10.16813/j.cnki.cn35-1286/g4.2017.05.023

[ref108] Lu Y. (2023) The relationship between natural connection and learning burnout among police academy students. Master’s thesis, People's Public Security University of China

[ref109] Lu P. Zhou J. (2019). An analysis of the current situation of academic burnout among vocational nursing students and its relationship with mobile phone dependence. Health Vocat. Educ. 37, 109–111.

[ref110] Ma Y. (2019) The relationship between learning burnout and mobile phone addiction in secondary vocational school students: the mediating role of academic self-efficacy. Master’s thesis, Northwest Normal University

[ref111] Ma A. Yang Y. Guo S. Li X. Zhang S. Chang H. (2022). Adolescent resilience and mobile phone addiction in Henan Province of China: impacts of chain mediating, coping style. PLoS One 17:e0278182. doi: 10.1371/journal.pone.0278182, 36574414 PMC9794036

[ref112] Ma J. Zhong Y. Liang H. Peng J. Duan B. Song Y. (2020). Study on interaction among mobile phone dependence, academic procrastination and learning burnout of nursing students in Guangzhou City. Occup and Health 36, 837–841. doi: 10.13329/j.cnki.zyyjk.2020.0223

[ref113] Madigan D. J. Curran T. (2021). Does burnout affect academic achievement? A meta-analysis of over 100,000 students. Educ. Psychol. Rev. 33, 387–405. doi: 10.1007/s10648-020-09533-1

[ref114] Mao P. Cai Z. Chen B. Sun X. (2024). The association between problematic internet use and burnout: a three-level meta-analysis. J. Affect. Disord. 352, 321–332. doi: 10.1016/j.jad.2024.01.240, 38302068

[ref115] Martin R. E. Ochsner K. N. (2016). The neuroscience of emotion regulation development: implications for education. Curr. Opin. Behav. Sci. 10, 142–148. doi: 10.1016/j.cobeha.2016.06.006, 27822488 PMC5096655

[ref116] Maslach C. Leiter M. (1997). The truth about Burnout. San Francisco: Josey Bass. Inc., Publishers.

[ref117] Maslach C. Schaufeli W. B. Leiter M. P. (2001). Job burnout. Annu. Rev. Psychol. 52, 397–422. doi: 10.1146/annurev.psych.52.1.397, 11148311

[ref118] Matthews M. Webb T. L. Roni S. Miranda S. Sheppes G. (2021). Identifying the determinants of emotion regulation choice: a systematic review with meta-analysis. Cogn. Emot. 35, 1056–1084. doi: 10.1080/02699931.2021.1945538, 34165040

[ref119] Mayerhofer D. Haider K. Amon M. Gächter A. O’Rourke T. Dale R. . (2024). The association between problematic smartphone use and mental health in Austrian adolescents and young adults. Healthcare 12:600. doi: 10.3390/healthcare12060600, 38540564 PMC10970667

[ref120] Meng X. Yu D. Chen Y. Zhang L. Fu X. (2023). Association between childhood maltreatment and empathy: a three-level meta-analytic review. Acta Psychol. Sin. 55, 1285–1300. doi: 10.3724/SP.J.1041.2023.01285

[ref9001] Moher D. Liberati A. Tetzlaff J. Altman DG. (2009). The PRISMA Group Preferred Reporting Items for Systematic Reviews and Meta-Analyses: The PRISMA Statement. PLOS Medicine. 6, e1000097. doi: 10.1371/journal.pmed.1000097, 19621072 PMC2707599

[ref9002] Moher D. Shamseer L. Clarke M. Ghersi D. Liberati A. Petticrew M. . (2015). Preferred reporting items for systematic review and meta-analysis protocols (PRISMA-P) 2015 statement. Systematic reviews, 4: 1. doi: 10.1186/2046-4053-4-1, 25554246 PMC4320440

[ref121] Nie X. (2014) Research on Smartphone Addiction and Sleep Quality and Learning Burnout of Secondary Vocational Students. [Master’s thesis, Zhengzhou University]

[ref122] Nong W. (2022). The relationship between mobile phone dependence and academic burnout among students in private undergraduate colleges in Guangxi: the mediating role of academic procrastination. Ability Wisdom 19, 169–172.

[ref123] O’Rourke M. Hammond S. O’Flynn S. Boylan G. (2010). The medical student stress profile: a tool for stress audit in medical training. Med. Educ. 44, 1027–1037. doi: 10.1111/j.1365-2923.2010.03734.x, 20880372

[ref124] Ong R. H. S. Sim H. S. Bergman M. M. How C. H. Png C. A. L. Lim C. S. . (2024). Prevalence and associations of problematic smartphone use with smartphone activities, psychological well-being, and sleep quality in a household survey of Singapore adults. PLoS One 19:e0315364. doi: 10.1371/journal.pone.0315364, 39693321 PMC11654946

[ref125] Panova T. Carbonell X. (2018). Is smartphone addiction really an addiction? J. Behav. Addict. 7, 252–259. doi: 10.1556/2006.7.2018.49, 29895183 PMC6174603

[ref126] Park N. Kim Y.-C. Shon H. Y. Shim H. (2013). Factors influencing smartphone use and dependency in South Korea. Comput. Hum. Behav. 29, 1763–1770. doi: 10.1016/j.chb.2013.02.008

[ref127] Park Y. Lee S. (2022). Gender differences in smartphone addiction and depression among Korean adolescents: focusing on the internal mechanisms of attention deficit and self-control. Comput. Hum. Behav. 136:107400. doi: 10.1016/j.chb.2022.107400

[ref128] Paterna A. Alcaraz-Ibáñez M. Aguilar-Parra J. M. Salavera C. Demetrovics Z. Griffiths M. D. (2024). Problematic smartphone use and academic achievement: a systematic review and meta-analysis. J. Behav. Addict. 13, 313–326. doi: 10.1556/2006.2024.00014, 38669081 PMC11220804

[ref129] Qin L. Chen S. Luo B. Chen Y. (2022). The effect of learning burnout on sleep quality in primary school students: the mediating role of mental health. Healthcare 10:2076. doi: 10.3390/healthcare10102076, 36292523 PMC9602333

[ref130] Qin P. Diao S. Li T. Huang M. Liu G. (2020). The effect of perceived stress on college students' mobile phone addiction: a serial mediation effect of self-control and learning burnout. J. Psychol. Sci. 43, 1111–1116. doi: 10.16719/j.cnki.1671-6981.20200512

[ref131] Qu X. Lu A. Song P. Lan Y. Cai R. (2017). The mechanism of mobile phone addiction influencing academic burnout with mediating effect of procrastination. Chin. J. Appl. Psychol. 23, 49–57.

[ref132] Rodgers M. A. Pustejovsky J. E. (2021). Evaluating meta-analytic methods to detect selective reporting in the presence of dependent effect sizes. Psychol. Methods 26, 141–160. doi: 10.1037/met0000300, 32673040

[ref133] Roig-Vila R. Prendes-Espinosa P. Urrea-Solano M. (2020). Problematic smartphone use in Spanish and Italian university students. Sustainability 12:10255. doi: 10.3390/su122410255

[ref134] Rozgonjuk D. Elhai J. D. Täht K. Vassil K. Levine J. C. Asmundson G. J. G. (2019). Non-social smartphone use mediates the relationship between intolerance of uncertainty and problematic smartphone use: evidence from a repeated-measures study. Comput. Hum. Behav. 96, 56–62. doi: 10.1016/j.chb.2019.02.013

[ref135] Rozgonjuk D. Saal K. Täht K. (2018). Problematic smartphone use, deep and surface approaches to learning, and social media use in lectures. Int. J. Environ. Res. Public Health 15:92. doi: 10.3390/ijerph15010092, 29316697 PMC5800191

[ref136] Samek D. R. Crumly B. Akua B. A. Dawson M. Duke-Marks A. (2024). Microaggressions, perceptions of campus climate, mental health, and alcohol use among first-year college students of color. J. Res. Adolesc. 34, 96–113. doi: 10.1111/jora.12897, 37984497

[ref137] Schaufeli W. B. Bakker A. B. (2004). Job demands, job resources, and their relationship with burnout and engagement: a multi-sample study. J. Organ. Behav. 25, 293–315. doi: 10.1002/job.248

[ref138] Schaufeli W. B. Martínez I. M. Pinto A. M. Salanova M. Bakker A. B. (2002). Burnout and engagement in university students: a cross-national study. J. Cross-Cult. Psychol. 33, 464–481. doi: 10.1177/0022022102033005003

[ref139] Shen Q. (2017). An empirical study on mobile phone dependence and its psychological correlates among vocational college students. J. High. Educ. 12, 31–33.

[ref140] Shi Y. (2023) The effect of mobile phone addiction on academic burnout in sports school graduate students: the mediating role of irrational procrastination. Master’s thesis, Guangzhou Sport University

[ref142] Su S. Pan T. Liu Q. Chen X. Wang Y. Li M. (2014). Development of the smartphone addiction scale for college students. Chin. Ment. Health J. 28, 392–397.

[ref143] Sunday O. J. Adesope O. O. Maarhuis P. L. (2021). The effects of smartphone addiction on learning: a meta-analysis. Comput. Hum. Behav. Rep. 4:100114. doi: 10.1016/j.chbr.2021.100114

[ref144] Tang L. Zhang F. Yin R. Fan Z. (2021). Effect of interventions on learning burnout: a systematic review and meta-analysis. Front. Psychol. 12:645662. doi: 10.3389/fpsyg.2021.645662, 33716914 PMC7952745

[ref145] Tomaszek K. Muchacka-Cymerman A. (2019). Sex differences in the relationship between student school burnout and problematic internet use among adolescents. Int. J. Environ. Res. Public Health 16:4107. doi: 10.3390/ijerph16214107, 31653105 PMC6862502

[ref146] Troll E. S. Friese M. Loschelder D. D. (2021). How students’ self-control and smartphone-use explain their academic performance. Comput. Hum. Behav. 117:106624. doi: 10.1016/j.chb.2020.106624

[ref147] Viechtbauer W. (2005). Bias and efficiency of meta-analytic variance estimators in the random-effects model. J. Educ. Behav. Stat. 30, 261–293. doi: 10.3102/10769986030003261

[ref148] Viechtbauer W. (2010). Conducting meta-analyses in R with the metafor package. J. Stat. Softw. 36, 1–48. doi: 10.18637/jss.v036.i03

[ref149] Von Keyserlingk L. Yamaguchi-Pedroza K. Arum R. Eccles J. S. (2022). Stress of university students before and after campus closure in response to COVID-19. J. Community Psychol. 50, 285–301. doi: 10.1002/jcop.22561, 33786864 PMC8250790

[ref150] Wacks Y. Weinstein A. M. (2021). Excessive smartphone use is associated with health problems in adolescents and young adults. Front. Psych. 12:669042. doi: 10.3389/fpsyt.2021.669042, 34140904 PMC8204720

[ref151] Wan J. (2020) Research on the Relationship among the Life Events, Mobile Phone Dependence, Emotional Intelligence and Academic Burnout of Adolescents. Master’s thesis, Xinyang Normal University

[ref153] Wang J. Bu L. Li Y. Song J. Li N. (2021). The mediating effect of academic engagement between psychological capital and academic burnout among nursing students during the COVID-19 pandemic: a cross-sectional study. Nurse Educ. Today 102:104938. doi: 10.1016/j.nedt.2021.104938, 33934039 PMC8669342

[ref154] Wang P. Lei L. Wang X. Nie J. Chu X. Jin S. (2018). The exacerbating role of perceived social support and the “buffering” role of depression in the relation between sensation seeking and adolescent smartphone addiction. Pers. Individ. Differ. 130, 129–134. doi: 10.1016/j.paid.2018.04.009

[ref155] Wang P. Liu S. Zhao M. Yang X. Zhang G. Chu X. . (2019). How is problematic smartphone use related to adolescent depression? A moderated mediation analysis. Child Youth Serv. Rev. 104:104384. doi: 10.1016/j.childyouth.2019.104384

[ref156] Wang X. Qiao Y. Wang S. (2023). Parental phubbing, problematic smartphone use, and adolescents' learning burnout: a cross-lagged panel analysis. J. Affect. Disord. 320, 442–449. doi: 10.1016/j.jad.2022.09.163, 36206880

[ref157] Wang X. Tan S. C. Li L. (2020). Technostress in university students’ technology-enhanced learning: an investigation from multidimensional person-environment misfit. Comput. Hum. Behav. 105:106208. doi: 10.1016/j.chb.2019.106208

[ref158] Wei H. Shi M. Li Y. (2023). Mediating effect of smartphone addiction on professional attitude and academic burnout of undergraduate nursing students. J. Jining Med. Univ. 46, 397–400.

[ref159] Wen F. Ding Y. Yang C. Ma S. Zhu J. Xiao H. . (2023). Influence of smartphone use motives on smartphone addiction during the COVID-19 epidemic in China: the moderating effect of age. Curr. Psychol. 42, 19316–19325. doi: 10.1007/s12144-022-03355-w, 35854703 PMC9282147

[ref160] Wickord L.-C. Quaiser-Pohl C. (2022). Psychopathological symptoms and personality traits as predictors of problematic smartphone use in different age groups. Behav. Sci. 12:20. doi: 10.3390/bs12020020, 35200272 PMC8869315

[ref161] Winskel H. Kim T.-H. Kardash L. Belic I. (2019). Smartphone use and study behavior: a Korean and Australian comparison. Heliyon 5:e02158. doi: 10.1016/j.heliyon.2019.e02158, 31384688 PMC6661454

[ref162] Wolotira E. A. (2023). Trauma, compassion fatigue, and burnout in nurses: the nurse leader's response. Nurse Lead. 21, 202–206. doi: 10.1016/j.mnl.2022.04.009, 35582625 PMC9098943

[ref163] Wong B. Y. Yeo K. J. Lee S.-H. (2024). Bibliometric analysis of smartphone addiction literature. SAGE Open 14:21582440241271286. doi: 10.1177/21582440241271286

[ref164] Wu R.-h. Chen S.-p. Ye Q.-y. Cai J.-y. Zhang J.-j. (2022). Analysis on effect of trait coping style between mobile phone dependence and learning burnout of college students. Occupat. Health 38, 1262–1266. doi: 10.13329/j.cnki.zyyjk.2022.0236

[ref165] Wu Y. Dai X. Y. Zhang J. (2007). Development of the student burnout inventory for junior middle school students. Chin. J. Clin. Psychol. 15, 118–120. doi: 10.16128/j.cnki.1005-3611.2007.02.005

[ref166] Wu C. Zhang Y. Huang S. Yuan Q. (2021). Does enterprise social media usage make the employee more productive? A meta-analysis. Telemat. Inform. 60:101578. doi: 10.1016/j.tele.2021.101578

[ref167] Xu B. Chen N.-S. Chen G. (2020). Effects of teacher role on student engagement in WeChat-based online discussion learning. Comput. Educ. 157:103956. doi: 10.1016/j.compedu.2020.103956

[ref168] Xue J. Wang J. Ma Z. Li C. He Y. (2022). The relationship between academic burnout and smartphone addiction among secondary vocational school students. IT CEO CIO Inform Times 9, 151–154.

[ref169] Yan Y.-W. Lin R.-M. Su Y.-K. Liu M.-Y. (2018). The relationship between adolescent academic stress and sleep quality: a multiple mediation model. Soc. Behav. Pers. 46, 63–77. doi: 10.2224/sbp.6530

[ref170] Yang Z. Asbury K. Griffiths M. D. (2019). An exploration of problematic smartphone use among Chinese university students: associations with academic anxiety, academic procrastination, self-regulation and subjective wellbeing. Int. J. Ment. Health Addict. 17, 596–614. doi: 10.1007/s11469-018-9961-1

[ref171] Yang G.-H. Cao X.-X. Fu Y.-Y. Wang N.-D. Lian S.-L. (2024). Mobile phone addiction and academic burnout: the mediating role of technology conflict and the protective role of mindfulness. Front. Psych. 15:1365914. doi: 10.3389/fpsyt.2024.1365914, 38501091 PMC10944904

[ref173] Yang L. Lian R. (2005). Current studies and prospects of learning burnout. J. Jimei Univ. 6, 54–58.

[ref174] Yang H. Liu B. Fang J. (2021). Stress and problematic smartphone use severity: smartphone use frequency and fear of missing out as mediators. Front. Psych. 12:659288. doi: 10.3389/fpsyt.2021.659288, 34140901 PMC8203830

[ref176] Yang X. Zhou Z. Liu Q. Fan C. (2019). Mobile phone addiction and adolescents’ anxiety and depression: the moderating role of mindfulness. J. Child Fam. Stud. 28, 822–830. doi: 10.1007/s10826-018-01323-2

[ref177] Yao W. Hou H. Yang P. Ni S. (2025). The co-occurrence of adolescent smartphone addiction and academic burnout: the role of smartphone stress and digital flourishing. Educ. Inf. Technol. 30, 4987–5007. doi: 10.1007/s10639-024-13017-y

[ref178] Ye X. (2021) Research on the effect of mobile phone dependence of left-behind children in rural areas on academic burnout. Master’s thesis, Jiangxi Agricultural University

[ref179] Ye Y. Huang X. Liu Y. (2021). Social support and academic burnout among university students: a moderated mediation model. Psychol. Res. Behav. Manag. 14, 335–344. doi: 10.2147/PRBM.S300797, 33776493 PMC7987309

[ref180] Ye X. Li Y. Liu Y. Zheng Q. Lin Z. Zeng Y. . (2023). Effect of fear of missing out on learning burnout in medical students: a moderated mediation. Front. Psych. 14:1289906. doi: 10.3389/fpsyt.2023.1289906, 38045622 PMC10690946

[ref181] Ying L. Biao S. Shaoying G. Xuechen D. Tingting P. (2016). Cultural differences on function of emotional expression suppression. Adv. Psychol. Sci. 24, 1647–1654. doi: 10.3724/sp.J.1042.2016.01647

[ref182] Yogesh M. Ladani H. Parmar D. (2024). Associations between smartphone addiction, parenting styles, and mental well-being among adolescents aged 15–19 years in Gujarat, India. BMC Public Health 24:2462. doi: 10.1186/s12889-024-19991-9, 39256701 PMC11385490

[ref183] Yu M. Chen S. Luo Y. Lai W. Zhang J. (2022). Relationship between academic burnout and mobile phone dependence among university students during COVID-19 prevention and control period. Strait J. Prevent. Med. 28, 35–38.

[ref184] Yu M. Yang L. Wu M. Bie D. (2023). The relationship between mobile phone dependence and learning burnout among college students under gender regulation. Adv. Educ. 13, 9016–9021.

[ref185] Yuan W. Ma L. (2024). Influence of mobile phone addiction on learning burnout of preschool education students in higher vocational colleges: the mediation of core self-evaluation. Campus Life Mental Health 22, 32–36. doi: 10.19521/j.cnki.1673-1662.2024.01.006

[ref186] Zhang R. (2017) A study of related factors of mobile phone dependence in high school students and the group psychological intervention. Master’s thesis, Shanxi Medical University

[ref187] Zhang C. (2021) A study on the current situation and influencing factors of academic burnout among medical students. Master’s thesis, Chongqing Medical University

[ref188] Zhang B. Cheng S. Zhang Y. Xiao W. (2019). Mobile phone addiction and learning burnout: the mediating effect of self-control. Chin. J. Health Psychol. 27, 435–438. doi: 10.13342/j.cnki.cjhp.2019.03.030

[ref189] Zhang Y. Gan Y. Cham H. (2007). Perfectionism, academic burnout and engagement among Chinese college students: a structural equation modeling analysis. Pers. Individ. Differ. 43, 1529–1540. doi: 10.1016/j.paid.2007.04.010

[ref190] Zhang H. Gao T. Hu Q. Zhao L. Wang X. Sun X. . (2023). Parental marital conflict, negative emotions, phubbing, and academic burnout among college students in the postpandemic era: a multiple mediating models. Psychol. Schs. 60, 1488–1498.

[ref191] Zhang C. Li G. Fan Z. Tang X. Zhang F. (2021). Psychological capital mediates the relationship between problematic smartphone use and learning burnout in Chinese medical undergraduates and postgraduates: a cross-sectional study. Front. Psychol. 12:600352. doi: 10.3389/fpsyg.2021.600352, 34054634 PMC8155251

[ref192] Zhang C.-H. Li G. Fan Z.-Y. Tang X.-J. Zhang F. (2021). Mobile phone addiction mediates the relationship between alexithymia and learning burnout in Chinese medical students: a structural equation model analysis. Psychol. Res. Behav. Manag. 14, 455–465. doi: 10.2147/PRBM.S304635, 33883952 PMC8053701

[ref193] Zhang Y. Liang H. Guo M. Wu Y. (2020). Anxiety and learning burnout: the mediating role of smartphone addiction. J. Mudanjiang Norm. Univ. 5, 138–144. doi: 10.13815/j.cnki.jmtc(pss).2020.05.015

[ref194] Zhang F. Ma C. Wang S. (2020). The impacts of university students’ learning lassitude and happiness on smartphone addiction in the mobile internet age. J. Shandong Univ. Technol. 36, 102–106.

[ref195] Zhang X. Shen Q. (2015). The relationship between mobile phone dependence and academic burnout: an empirical study of vocational college students. Career Horizon 11, 96–99.

[ref196] Zhang W. Wu P. Zheng X. Guo F. (2023). The relationships among mobile phone dependence, time management disposition, and academic burnout in nursing students at vocational colleges. Estate Sci Tribune 22, 84–85.

[ref197] Zhang M. Xu W. Zhou H. Fan J. Liu H. (2024). Impact of COVID-19 on academic burnout among medical college students in China: findings from a web-based survey. Med. Sci. Monit. 30:e942317. doi: 10.12659/MSM.942317, 38291742 PMC10840366

[ref198] Zhao X. (2024) The study on the influencing factors and mechanism path of problematic smartphone use among adolescents. Master’s thesis, Jilin University

[ref199] Zhong Y. Ma H. Liang Y.-F. Liao C.-J. Zhang C.-C. Jiang W.-J. (2022). Prevalence of smartphone addiction among Asian medical students: a meta-analysis of multinational observational studies. Int. J. Soc. Psychiatry 68, 1171–1183. doi: 10.1177/00207640221089535, 35422151

[ref200] Zhou Q. (2021) The relationship among mobile phone addiction, psychological capital and academic burnout of college students and intervention research. Master’s thesis, Hebei Normal University

[ref201] Zhou Z. Liu H. Zhang D. Wei H. Zhang M. Huang A. (2022). Mediating effects of academic self-efficacy and smartphone addiction on the relationship between professional attitude and academic burnout in nursing students: a cross-sectional study. Nurse Educ. Today 116:105471. doi: 10.1016/j.nedt.2022.105471, 35834868

[ref202] Zhu L. Hou J. Zhou B. Xiao X. Wang J. Jia W. (2023). Physical activity, problematic smartphone use, and burnout among Chinese college students. PeerJ 11:e16270. doi: 10.7717/peerj.16270, 37842034 PMC10576493

[ref203] Zou L. (2018) A study on the relationship between mobile phone dependence, social support, and academic burnout among junior high school students. Master’s thesis, Mudanjiang Normal University

[ref204] Zou J. (2019) The influence of parental education level on adolescents' attention, executive function, and academic performance. Master's thesis, Shaanxi Normal University

