# Supplementary material for: The relationship between academic burnout and problematic smartphone use: a three-level meta-analysis
Source: Front Psychol. 2026 Apr 2;17:1768092. doi: 10.3389/fpsyg.2026.1768092 (PMC13083086; doi:10.3389/fpsyg.2026.1768092)
Supplement: Supplementary file 1 [file Table_1.DOCX]

| **Supplementary Material**  **Table S1**. Quality Assessment of Primary Studies Included in the Meta-analysis   \| ID \| Author (Year) \| Item 1 \| Item 2 \| Item 3 \| Item 4 \| Item 5 \| Item 6 \| Item 7 \| Item 8 \| Item 9 \| Item 10 \| Item 11 \| Item 12 \| Item 13 \| Item 14 \| Points \| \| --- \| --- \| --- \| --- \| --- \| --- \| --- \| --- \| --- \| --- \| --- \| --- \| --- \| --- \| --- \| --- \| --- \| \| 1 \| Bai et al. (2020) \| Y \| Y \| Y \| Y \| N \| N \| N \| Y \| Y \| N \| Y \| N \| NA \| N \| 7 \| \| 2 \| Cheng and Zhang (2020) \| Y \| Y \| Y \| N \| N \| N \| N \| Y \| Y \| N \| Y \| N \| NA \| N \| 6 \| \| 3 \| Chen et al. (2023) \| Y \| Y \| Y \| Y \| N \| N \| N \| Y \| Y \| N \| Y \| N \| NA \| N \| 7 \| \| 4 \| Hao et al. (2021) \| Y \| Y \| Y \| Y \| N \| N \| N \| Y \| Y \| N \| Y \| N \| NA \| Y \| 8 \| \| 5 \| Hao et al. (2022) \| Y \| Y \| Y \| Y \| N \| N \| N \| Y \| Y \| N \| Y \| N \| NA \| Y \| 8 \| \| 6 \| Hu et al. (2024) \| Y \| Y \| Y \| Y \| N \| N \| N \| Y \| Y \| Y \| Y \| N \| NA \| Y \| 9 \| \| 7 \| Jiang et al. (2024) \| Y \| Y \| Y \| Y \| N \| N \| N \| Y \| Y \| N \| Y \| N \| NA \| Y \| 8 \| \| 8 \| Jin et al. (2024) \| Y \| Y \| Y \| Y \| N \| N \| N \| Y \| Y \| N \| Y \| N \| NA \| N \| 7 \| \| 9 \| Kaya (2024) \| Y \| Y \| Y \| Y \| Y \| N \| N \| Y \| Y \| N \| Y \| N \| NA \| Y \| 9 \| \| 10 \| Li, Xu, et al. (2024) \| Y \| Y \| Y \| Y \| Y \| N \| N \| Y \| Y \| N \| Y \| N \| NA \| Y \| 9 \| \| 11 \| Li et al. (2021) \| Y \| Y \| Y \| Y \| Y \| N \| N \| Y \| Y \| N \| Y \| N \| NA \| Y \| 9 \| \| 12 \| Liu (2023) \| Y \| Y \| Y \| Y \| N \| Y \| Y \| Y \| Y \| Y \| Y \| N \| NA \| N \| 10 \| \| 13 \| Qin et al. (2020) \| Y \| Y \| Y \| Y \| N \| N \| N \| Y \| Y \| N \| Y \| N \| NA \| N \| 7 \| \| 14 \| Samek et al. (2024) \| Y \| Y \| Y \| Y \| N \| N \| N \| Y \| Y \| N \| Y \| N \| NA \| N \| 7 \| \| 15 \| Wang et al. (2023) \| Y \| Y \| Y \| Y \| N \| N \| Y \| Y \| Y \| Y \| Y \| N \| NA \| Y \| 10 \| \| 16 \| Yang et al. (2024) \| Y \| Y \| Y \| Y \| N \| N \| N \| Y \| Y \| N \| Y \| N \| NA \| Y \| 8 \| \| 17 \| Yao et al. (2025) \| Y \| Y \| Y \| Y \| Y \| N \| N \| Y \| Y \| N \| Y \| N \| NA \| Y \| 9 \| \| 18 \| Ye et al. (2023) \| Y \| Y \| Y \| Y \| N \| N \| N \| Y \| Y \| N \| Y \| N \| NA \| N \| 7 \| \| 19 \| Zhang et al. (2021A) \| Y \| Y \| Y \| N \| N \| N \| N \| Y \| Y \| N \| Y \| N \| NA \| Y \| 7 \| \| 20 \| Zhang et al. (2021B) \| Y \| Y \| Y \| N \| Y \| N \| N \| Y \| Y \| N \| Y \| N \| NA \| Y \| 8 \| \| 21 \| Zhang, Gao, et al. (2023) \| Y \| Y \| Y \| Y \| N \| N \| N \| Y \| Y \| N \| Y \| N \| NA \| N \| 7 \| \| 22 \| Zhang et al. (2024) \| Y \| Y \| Y \| Y \| N \| N \| N \| Y \| Y \| N \| Y \| N \| NA \| N \| 7 \| \| 23 \| Zhou et al. (2022) \| Y \| Y \| Y \| N \| N \| N \| N \| Y \| Y \| N \| Y \| N \| NA \| N \| 6 \| \| 24 \| Zhu et al. (2023) \| Y \| Y \| Y \| Y \| Y \| N \| N \| Y \| Y \| N \| Y \| N \| NA \| Y \| 9 \| \| 25 \| Wan (2020) \| Y \| Y \| Y \| Y \| N \| Y \| Y \| Y \| Y \| Y \| Y \| N \| NA \| Y \| 11 \| \| 26 \| He et al. (2022) \| Y \| Y \| Y \| N \| N \| N \| N \| Y \| Y \| N \| Y \| N \| NA \| N \| 6 \| \| 27 \| Yu et al. (2022) \| Y \| Y \| Y \| Y \| N \| N \| N \| Y \| Y \| N \| Y \| N \| NA \| N \| 7 \| \| 28 \| (Nong, 2022) \| Y \| Y \| Y \| Y \| N \| N \| N \| Y \| Y \| N \| Y \| N \| NA \| Y \| 8 \| \| 29 \| Feng and Tao (2019) \| Y \| Y \| Y \| Y \| N \| N \| N \| Y \| Y \| N \| Y \| N \| NA \| Y \| 8 \| \| 30 \| Liu, Wang, et al. (2022) \| Y \| Y \| Y \| Y \| N \| N \| N \| Y \| Y \| N \| Y \| N \| NA \| N \| 7 \| \| 31 \| Liu et al. (2019) \| Y \| Y \| Y \| N \| N \| N \| N \| Y \| Y \| N \| Y \| N \| NA \| N \| 6 \| \| 32 \| Liu et al. (2021) \| Y \| Y \| Y \| Y \| N \| N \| N \| Y \| Y \| N \| Y \| N \| NA \| N \| 7 \| \| 33 \| Lu (2017) \| Y \| Y \| Y \| Y \| N \| N \| N \| Y \| Y \| N \| Y \| N \| NA \| Y \| 8 \| \| 34 \| Wu et al. (2022) \| Y \| Y \| Y \| Y \| N \| N \| N \| Y \| Y \| N \| Y \| N \| NA \| N \| 7 \| \| 35 \| Zhou (2021) \| Y \| Y \| Y \| Y \| N \| N \| N \| Y \| Y \| N \| Y \| N \| NA \| N \| 7 \| \| 36 \| Cui (2023) \| Y \| Y \| Y \| Y \| N \| N \| N \| Y \| Y \| N \| Y \| N \| NA \| N \| 7 \| \| 37 \| Zhang, Wu, et al. (2023) \| Y \| Y \| Y \| N \| N \| N \| N \| Y \| Y \| N \| Y \| N \| NA \| N \| 6 \| \| 38 \| Zhang and Shen (2015) \| Y \| Y \| Y \| N \| N \| N \| N \| Y \| Y \| N \| Y \| N \| NA \| N \| 6 \| \| 39 \| Zhang et al. (2019) \| Y \| Y \| Y \| N \| N \| N \| N \| Y \| Y \| N \| Y \| N \| NA \| N \| 6 \| \| 40 \| Zhang et al. (2020A) \| Y \| Y \| Y \| Y \| N \| N \| N \| Y \| Y \| N \| Y \| N \| NA \| N \| 7 \| \| 41 \| Zhang et al. (2020B) \| Y \| Y \| Y \| Y \| N \| N \| N \| Y \| Y \| N \| Y \| N \| NA \| N \| 7 \| \| 42 \| Zhang (2017) \| Y \| Y \| Y \| Y \| N \| N \| N \| Y \| Y \| N \| Y \| N \| NA \| N \| 7 \| \| 43 \| Zhang (2021) \| Y \| Y \| Y \| N \| N \| N \| N \| Y \| Y \| N \| Y \| N \| NA \| N \| 6 \| \| 44 \| Qu et al. (2017) \| Y \| Y \| Y \| Y \| N \| N \| N \| Y \| Y \| N \| Y \| N \| NA \| Y \| 8 \| \| 45 \| Cao (2018) \| Y \| Y \| Y \| Y \| N \| N \| N \| Y \| Y \| N \| Y \| N \| NA \| N \| 7 \| \| 46 \| Li et al. (2022A) \| Y \| Y \| Y \| N \| N \| N \| N \| Y \| Y \| N \| Y \| N \| NA \| N \| 6 \| \| 47 \| Li et al. (2022B) \| Y \| Y \| Y \| Y \| N \| N \| N \| Y \| Y \| N \| Y \| N \| NA \| N \| 7 \| \| 48 \| Liang (2019) \| Y \| Y \| Y \| Y \| N \| N \| N \| Y \| Y \| N \| Y \| N \| NA \| N \| 7 \| \| 49 \| Shen (2017) \| Y \| Y \| Y \| N \| N \| N \| N \| Y \| Y \| N \| Y \| N \| NA \| N \| 6 \| \| 50 \| Wang (2020) \| Y \| Y \| Y \| N \| N \| N \| N \| Y \| Y \| N \| Y \| N \| NA \| Y \| 7 \| \| 51 \| Cheng (2021) \| Y \| Y \| Y \| Y \| N \| N \| N \| Y \| Y \| N \| Y \| N \| NA \| Y \| 8 \| \| 52 \| Cheng (2019) \| Y \| Y \| Y \| N \| N \| N \| N \| Y \| Y \| N \| Y \| N \| NA \| Y \| 7 \| \| 53 \| Nie (2014) \| Y \| Y \| Y \| Y \| N \| N \| N \| Y \| Y \| N \| Y \| N \| NA \| Y \| 8 \| \| 54 \| Hu (2022) \| Y \| Y \| Y \| Y \| N \| N \| N \| Y \| Y \| N \| Y \| N \| NA \| Y \| 8 \| \| 55 \| Ge (2013) \| Y \| Y \| Y \| Y \| N \| N \| N \| Y \| Y \| N \| Y \| N \| NA \| N \| 7 \| \| 56 \| Jiang et al. (2017) \| Y \| Y \| Y \| N \| N \| N \| N \| Y \| Y \| N \| Y \| N \| NA \| N \| 6 \| \| 57 \| Xue et al. (2022) \| Y \| Y \| Y \| Y \| N \| N \| N \| Y \| Y \| N \| Y \| N \| NA \| N \| 7 \| \| 58 \| Yuan and Ma (2024) \| Y \| Y \| Y \| Y \| N \| N \| N \| Y \| Y \| N \| Y \| N \| NA \| N \| 7 \| \| 59 \| Zhao (2024) \| Y \| Y \| Y \| Y \| N \| N \| N \| Y \| Y \| N \| Y \| N \| NA \| Y \| 8 \| \| 60 \| Deng (2021) \| Y \| Y \| Y \| N \| N \| N \| N \| Y \| Y \| N \| Y \| N \| NA \| N \| 6 \| \| 61 \| Zou (2018) \| Y \| Y \| Y \| Y \| N \| N \| N \| Y \| Y \| N \| Y \| N \| NA \| N \| 7 \| \| 62 \| Lu and Zhou (2019) \| Y \| Y \| Y \| N \| N \| N \| N \| Y \| Y \| N \| Y \| N \| NA \| N \| 6 \| \| 63 \| Lu (2023) \| Y \| Y \| Y \| Y \| N \| N \| N \| Y \| Y \| N \| Y \| N \| NA \| N \| 7 \| \| 64 \| Chen et al. (2022) \| Y \| Y \| Y \| Y \| N \| N \| N \| Y \| Y \| N \| Y \| N \| NA \| N \| 7 \| \| 65 \| Chen et al. (2024) \| Y \| Y \| Y \| N \| N \| N \| N \| Y \| Y \| N \| Y \| N \| NA \| N \| 6 \| \| 66 \| Chen et al. (2021) \| Y \| Y \| Y \| N \| N \| N \| N \| Y \| Y \| N \| Y \| N \| NA \| N \| 6 \| \| 67 \| Chen (2019) \| Y \| Y \| Y \| Y \| N \| N \| N \| Y \| Y \| N \| Y \| N \| NA \| N \| 7 \| \| 68 \| Li et al. (2022) \| Y \| Y \| Y \| Y \| N \| N \| N \| Y \| Y \| N \| Y \| N \| NA \| N \| 7 \| \| 69 \| Wei et al. (2023) \| Y \| Y \| Y \| N \| N \| N \| N \| Y \| Y \| N \| Y \| N \| NA \| N \| 6 \| \| 70 \| Gu et al. (2021) \| Y \| Y \| Y \| N \| N \| N \| N \| Y \| Y \| N \| Y \| N \| NA \| N \| 6 \| \| 71 \| Ma et al. (2020) \| Y \| Y \| Y \| Y \| N \| N \| N \| Y \| Y \| N \| Y \| N \| NA \| N \| 7 \| \| 72 \| Ma (2019) \| Y \| Y \| Y \| N \| N \| N \| N \| Y \| Y \| N \| Y \| N \| NA \| Y \| 7 \| \| 73 \| Huang and Zhou (2016) \| Y \| Y \| Y \| N \| N \| N \| N \| Y \| Y \| N \| Y \| N \| NA \| N \| 6 \| \| 74 \| Ye (2021) \| Y \| Y \| Y \| Y \| N \| N \| N \| Y \| Y \| N \| Y \| N \| NA \| N \| 7 \| \| 75 \| Li et al. (2020) \| Y \| Y \| Y \| N \| N \| N \| N \| Y \| Y \| N \| Y \| N \| NA \| N \| 6 \| \| 76 \| Liu and Jin (2018) \| Y \| Y \| Y \| Y \| N \| N \| N \| Y \| Y \| N \| Y \| N \| NA \| N \| 7 \| \| 77 \| Yu et al. (2023) \| Y \| Y \| Y \| N \| N \| N \| N \| Y \| Y \| N \| Y \| N \| NA \| N \| 6 \| \| 78 \| Cheng et al. (2018) \| Y \| Y \| Y \| Y \| N \| N \| N \| Y \| Y \| N \| Y \| N \| NA \| Y \| 8 \| \| 79 \| Shi (2023) \| Y \| Y \| Y \| Y \| N \| N \| N \| Y \| Y \| N \| Y \| N \| NA \| N \| 7 \| |  |  |  |  |  |  |  |  |  |  |  |  |  |  |  |  |
| --- | --- | --- | --- | --- | --- | --- | --- | --- | --- | --- | --- | --- | --- | --- | --- | --- | --- | --- | --- | --- | --- | --- | --- | --- | --- | --- | --- | --- | --- | --- | --- | --- | --- | --- | --- | --- | --- | --- | --- | --- | --- | --- | --- | --- | --- | --- | --- | --- | --- | --- | --- | --- | --- | --- | --- | --- | --- | --- | --- | --- | --- | --- | --- | --- | --- | --- | --- | --- | --- | --- | --- | --- | --- | --- | --- | --- | --- | --- | --- | --- | --- | --- | --- | --- | --- | --- | --- | --- | --- | --- | --- | --- | --- | --- | --- | --- | --- | --- | --- | --- | --- | --- | --- | --- | --- | --- | --- | --- | --- | --- | --- | --- | --- | --- | --- | --- | --- | --- | --- | --- | --- | --- | --- | --- | --- | --- | --- | --- | --- | --- | --- | --- | --- | --- | --- | --- | --- | --- | --- | --- | --- | --- | --- | --- | --- | --- | --- | --- | --- | --- | --- | --- | --- | --- | --- | --- | --- | --- | --- | --- | --- | --- | --- | --- | --- | --- | --- | --- | --- | --- | --- | --- | --- | --- | --- | --- | --- | --- | --- | --- | --- | --- | --- | --- | --- | --- | --- | --- | --- | --- | --- | --- | --- | --- | --- | --- | --- | --- | --- | --- | --- | --- | --- | --- | --- | --- | --- | --- | --- | --- | --- | --- | --- | --- | --- | --- | --- | --- | --- | --- | --- | --- | --- | --- | --- | --- | --- | --- | --- | --- | --- | --- | --- | --- | --- | --- | --- | --- | --- | --- | --- | --- | --- | --- | --- | --- | --- | --- | --- | --- | --- | --- | --- | --- | --- | --- | --- | --- | --- | --- | --- | --- | --- | --- | --- | --- | --- | --- | --- | --- | --- | --- | --- | --- | --- | --- | --- | --- | --- | --- | --- | --- | --- | --- | --- | --- | --- | --- | --- | --- | --- | --- | --- | --- | --- | --- | --- | --- | --- | --- | --- | --- | --- | --- | --- | --- | --- | --- | --- | --- | --- | --- | --- | --- | --- | --- | --- | --- | --- | --- | --- | --- | --- | --- | --- | --- | --- | --- | --- | --- | --- | --- | --- | --- | --- | --- | --- | --- | --- | --- | --- | --- | --- | --- | --- | --- | --- | --- | --- | --- | --- | --- | --- | --- | --- | --- | --- | --- | --- | --- | --- | --- | --- | --- | --- | --- | --- | --- | --- | --- | --- | --- | --- | --- | --- | --- | --- | --- | --- | --- | --- | --- | --- | --- | --- | --- | --- | --- | --- | --- | --- | --- | --- | --- | --- | --- | --- | --- | --- | --- | --- | --- | --- | --- | --- | --- | --- | --- | --- | --- | --- | --- | --- | --- | --- | --- | --- | --- | --- | --- | --- | --- | --- | --- | --- | --- | --- | --- | --- | --- | --- | --- | --- | --- | --- | --- | --- | --- | --- | --- | --- | --- | --- | --- | --- | --- | --- | --- | --- | --- | --- | --- | --- | --- | --- | --- | --- | --- | --- | --- | --- | --- | --- | --- | --- | --- | --- | --- | --- | --- | --- | --- | --- | --- | --- | --- | --- | --- | --- | --- | --- | --- | --- | --- | --- | --- | --- | --- | --- | --- | --- | --- | --- | --- | --- | --- | --- | --- | --- | --- | --- | --- | --- | --- | --- | --- | --- | --- | --- | --- | --- | --- | --- | --- | --- | --- | --- | --- | --- | --- | --- | --- | --- | --- | --- | --- | --- | --- | --- | --- | --- | --- | --- | --- | --- | --- | --- | --- | --- | --- | --- | --- | --- | --- | --- | --- | --- | --- | --- | --- | --- | --- | --- | --- | --- | --- | --- | --- | --- | --- | --- | --- | --- | --- | --- | --- | --- | --- | --- | --- | --- | --- | --- | --- | --- | --- | --- | --- | --- | --- | --- | --- | --- | --- | --- | --- | --- | --- | --- | --- | --- | --- | --- | --- | --- | --- | --- | --- | --- | --- | --- | --- | --- | --- | --- | --- | --- | --- | --- | --- | --- | --- | --- | --- | --- | --- | --- | --- | --- | --- | --- | --- | --- | --- | --- | --- | --- | --- | --- | --- | --- | --- | --- | --- | --- | --- | --- | --- | --- | --- | --- | --- | --- | --- | --- | --- | --- | --- | --- | --- | --- | --- | --- | --- | --- | --- | --- | --- | --- | --- | --- | --- | --- | --- | --- | --- | --- | --- | --- | --- | --- | --- | --- | --- | --- | --- | --- | --- | --- | --- | --- | --- | --- | --- | --- | --- | --- | --- | --- | --- | --- | --- | --- | --- | --- | --- | --- | --- | --- | --- | --- | --- | --- | --- | --- | --- | --- | --- | --- | --- | --- | --- | --- | --- | --- | --- | --- | --- | --- | --- | --- | --- | --- | --- | --- | --- | --- | --- | --- | --- | --- | --- | --- | --- | --- | --- | --- | --- | --- | --- | --- | --- | --- | --- | --- | --- | --- | --- | --- | --- | --- | --- | --- | --- | --- | --- | --- | --- | --- | --- | --- | --- | --- | --- | --- | --- | --- | --- | --- | --- | --- | --- | --- | --- | --- | --- | --- | --- | --- | --- | --- | --- | --- | --- | --- | --- | --- | --- | --- | --- | --- | --- | --- | --- | --- | --- | --- | --- | --- | --- | --- | --- | --- | --- | --- | --- | --- | --- | --- | --- | --- | --- | --- | --- | --- | --- | --- | --- | --- | --- | --- | --- | --- | --- | --- | --- | --- | --- | --- | --- | --- | --- | --- | --- | --- | --- | --- | --- | --- | --- | --- | --- | --- | --- | --- | --- | --- | --- | --- | --- | --- | --- | --- | --- | --- | --- | --- | --- | --- | --- | --- | --- | --- | --- | --- | --- | --- | --- | --- | --- | --- | --- | --- | --- | --- | --- | --- | --- | --- | --- | --- | --- | --- | --- | --- | --- | --- | --- | --- | --- | --- | --- | --- | --- | --- | --- | --- | --- | --- | --- | --- | --- | --- | --- | --- | --- | --- | --- | --- | --- | --- | --- | --- | --- | --- | --- | --- | --- | --- | --- | --- | --- | --- | --- | --- | --- | --- | --- | --- | --- | --- | --- | --- | --- | --- | --- | --- | --- | --- | --- | --- | --- | --- | --- | --- | --- | --- | --- | --- | --- | --- | --- | --- | --- | --- | --- | --- | --- | --- | --- | --- | --- | --- | --- | --- | --- | --- | --- | --- | --- | --- | --- | --- | --- | --- | --- | --- | --- | --- | --- | --- | --- | --- | --- | --- | --- | --- | --- | --- | --- | --- | --- | --- | --- | --- | --- | --- | --- | --- | --- | --- | --- | --- | --- | --- | --- | --- | --- | --- | --- | --- | --- | --- | --- | --- | --- | --- | --- | --- | --- | --- | --- | --- | --- | --- | --- | --- | --- | --- | --- | --- | --- | --- | --- | --- | --- | --- | --- | --- | --- | --- | --- | --- | --- | --- | --- | --- | --- | --- | --- | --- | --- | --- | --- | --- | --- | --- | --- | --- | --- | --- | --- | --- | --- | --- | --- | --- | --- | --- | --- | --- | --- | --- | --- | --- | --- | --- | --- | --- | --- | --- | --- | --- | --- | --- | --- | --- | --- | --- | --- | --- | --- | --- | --- | --- | --- | --- | --- | --- | --- | --- | --- | --- | --- | --- | --- | --- | --- | --- | --- | --- | --- | --- | --- | --- | --- | --- | --- | --- | --- | --- | --- | --- | --- | --- | --- | --- | --- | --- | --- | --- | --- | --- | --- | --- | --- | --- | --- | --- | --- | --- | --- | --- | --- | --- | --- | --- | --- | --- | --- | --- | --- | --- | --- | --- | --- | --- | --- | --- | --- | --- | --- | --- | --- | --- | --- | --- | --- | --- | --- | --- | --- | --- | --- | --- | --- | --- | --- | --- | --- | --- | --- | --- | --- | --- | --- | --- | --- | --- | --- | --- | --- | --- | --- | --- | --- | --- | --- | --- | --- | --- | --- | --- | --- | --- | --- | --- | --- | --- | --- | --- | --- | --- | --- | --- | --- | --- | --- | --- | --- | --- | --- | --- | --- | --- | --- | --- | --- | --- | --- | --- | --- | --- | --- | --- | --- | --- | --- | --- | --- | --- | --- | --- | --- | --- | --- | --- | --- | --- | --- | --- | --- | --- | --- | --- | --- | --- | --- | --- | --- | --- | --- | --- | --- | --- | --- | --- | --- | --- | --- | --- | --- | --- | --- | --- | --- | --- | --- | --- | --- | --- | --- | --- | --- | --- | --- | --- | --- | --- | --- | --- | --- | --- | --- | --- | --- | --- | --- | --- | --- | --- | --- | --- | --- | --- | --- | --- | --- | --- | --- | --- | --- | --- | --- | --- | --- | --- | --- | --- | --- | --- | --- | --- | --- | --- | --- | --- | --- | --- | --- | --- | --- | --- | --- | --- | --- | --- | --- | --- | --- | --- | --- | --- | --- | --- | --- | --- | --- | --- | --- | --- | --- | --- | --- | --- | --- | --- | --- | --- | --- | --- | --- | --- | --- | --- | --- | --- | --- | --- | --- | --- | --- | --- | --- | --- | --- |

Note: The quality of each study was evaluated using the Quality Assessment Tool for Observational Cohort and Cross-Sectional Studies developed by the National Institutes of Health (NIH).

1. Was the research question or objective in this paper clearly stated?

2. Was the study population clearly specified and defined?

3. Was the participation rate of eligible persons at least 50%?

4. Were all the subjects selected or recruited from the same or similar populations (including the same time period)? Were inclusion and exclusion criteria for being in the study prespecified and applied uniformly to all participants?

5. Was a sample size justification, power description, or variance and effect estimates provided?

6. For the analyses in this paper, were the exposure(s)of interest measured prior to the outcome(s)being measured?

7. Was the timeframe sufficient so that one could reasonably expect to see an association between exposure and outcome if it existed?

8. For exposures that can vary in amount or level, did the study examine different levels of the exposure as related to the outcome (e.g., categories of exposure, or exposure measured as continuous variable)?

9. Were the exposure measures(independent variables)clearly defined, valid, reliable, and implemented consistently across all study participants?

10. Was the exposure(s)assessed more than once over time?

11. Were the outcome measures (dependent variables) clearly defined, valid, reliable, and implemented consistently across all study participants?

12. Were the outcome assessors blinded to the exposure status of participants?

13. Was loss to follow-up after baseline 20%or less?

14. Were key potential confounding variables measured and adjusted statistically for their impact on the relationship between exposure(s)and outcome(s)?

The quality score for each study was calculated by summing the responses to all 14 criteria (1 point for “Yes”; 0 points for “No” or “Not Applicable”). **Y = Yes, N = No, NA = Not Applicable.**
